# Disrupted expression of mitochondrial NCLX sensitizes neuroglial networks to excitotoxic stimuli and renders synaptic activity toxic

**DOI:** 10.1016/j.jbc.2021.101508

**Published:** 2021-12-20

**Authors:** Anna M. Hagenston, Jing Yan, Carlos Bas-Orth, Yanwei Tan, Israel Sekler, Hilmar Bading

**Affiliations:** 1 Department of Neurobiology, Interdisciplinary Center for Neurosciences, Heidelberg University, Heidelberg, Germany; 2 Department of Physiology and Cell Biology, Faculty of Health Sciences, Ben-Gurion University of the Negev, Beer-Sheva, Israel

**Keywords:** calcium signaling, NCLX, mitochondria, gene expression, neurotoxicity, synaptic activity, *Actb*, actin beta, AD, Alzheimer's disease, *Aqp4*, aquaporin 4, AraC, cytosine arabinoside, *Arc*, activity regulated cytoskeletal-associated protein, *Atf3*, activating transcription factor 3, *Bdnf*, brain derived neurotrophic factor, CA1, cornu ammonis 1, CaMK2a, calcium/calmodulin-dependent protein kinase II alpha, *cFos*, FBJ osteosarcoma oncogene, DFG, Deutsche Forschungsgemeinschaft, DIV, day in vitro, EGFP, enhanced GFP, FCCP, carbonyl cyanide-*p*-trifluoromethoxyphenylhydrazone, FJC, Fluoro-Jade C, FRET, Förster resonance energy transfer, GABA_A_R, gamma-aminobutyric acid (GABA) A receptor, GFAP, glial fibrillary acidic protein, *Gusb*, glucoronidase, beta, HBS, Hepes-buffered saline, HEK293, human embryonic kidney 293 cells, ITR, inverted terminal repeat, MCU, mitochondrial calcium uniporter, *Meg3*, maternally expressed 3, *Micu1*, mitochondrial calcium uptake 1, *mt-Atp6*, ATP synthase F0 subunit 6, mitochondrial, *mt-Co1*, cytochrome c oxidase I, mitochondrial, *mt-Co2*, cytochrome c oxidase II, mitochondrial, *mt-Nd1*, NADH dehydrogenase I, mitochondrial, NCLX, solute carrier family 8 sodium/calcium/lithium exchanger, member B1, NMDA, *N*-methyl-d-aspartate, NMDAR, *N*-methyl-d-aspartate receptor, *Npas4*, neuronal PAS domain protein 4, pCAG, CAG promoter, pCaMK2a, calcium/calmodulin-dependent protein kinase II alpha promoter, PD, Parkinson's disease, *Ppargc1a*, peroxisome proliferative activated receptor, gamma, coactivator 1 alpha, pU6, U6 small nuclear RNA promoter, qRT–PCR, quantitative reverse transcription polymerase chain reaction, rAAV, recombinant adeno-associated viral vector, Rh123, rhodamine 123, ROI, region of interest, ROS, reactive oxygen species, RRID, Research Resource Identifier, Ru360, ruthenium 360, shRNA, short hairpin RNA, *Tfam*, transcription factor A, mitochondrial, WPRE, woodchuck hepatitis virus posttranscriptional regulatory element, ΔΨ_m_, mitochondrial membrane potential, 3′UTR, 3′ untranslated region, *Vdac1*, voltage-dependent anion channel 1

## Abstract

The mitochondrial solute carrier family 8 sodium/calcium/lithium exchanger, member B1 (NCLX) is an important mediator of calcium extrusion from mitochondria. In this study, we tested the hypothesis that physiological expression levels of NCLX are essential for maintaining neuronal resilience in the face of excitotoxic challenge. Using an shRNA-mediated approach, we showed that reduced NCLX expression exacerbates neuronal mitochondrial calcium dysregulation, mitochondrial membrane potential (ΔΨ_m_) breakdown, and reactive oxygen species generation during excitotoxic stimulation of primary hippocampal cultures. Moreover, NCLX knockdown—which affected both neurons and glia—resulted not only in enhanced neurodegeneration following an excitotoxic insult but also in neuronal and astrocytic cell death under basal conditions. Our data also revealed that synaptic activity, which promotes neuroprotective signaling, can become lethal upon NCLX depletion; expression of NCLX-targeted shRNA impaired the clearance of mitochondrial calcium following action potential bursts, and was associated both with ΔΨ_m_ breakdown and substantial neurodegeneration in hippocampal cultures undergoing synaptic activity. Finally, we showed that NCLX knockdown within the hippocampal cornu ammonis 1 region *in vivo* causes substantial neurodegeneration and astrodegeneration. In summary, we demonstrated that dysregulated NCLX expression not only sensitizes neuroglial networks to excitotoxic stimuli but also notably renders otherwise neuroprotective synaptic activity toxic. These findings may explain the emergence of neurodegeneration and astrodegeneration in patients with disorders characterized by disrupted NCLX expression or function, and suggest that treatments aimed at enhancing or restoring NCLX function may prevent central nervous system damage in these disease states.

Mitochondrial dysfunction in general—and disturbed mitochondrial calcium signaling in particular—has been linked to death processes in numerous cell types from tissues throughout the body, including the central and peripheral nervous systems (1, 2, 3). Mitochondrial calcium dyshomeostasis is particularly relevant in the mechanisms underlying excitotoxic cell death and may represent the common denominator triggering cellular loss in a wide range of acute and chronic neurological diseases with excitotoxic components. Indeed, a cascade of events involving extrasynaptic *N*-methyl-d-aspartate receptor (NMDAR)-dependent calcium entry, mitochondrial calcium overload, breakdown of the mitochondrial membrane potential (ΔΨ_m_), disrupted energy metabolism, mitochondrial permeability transition, and ultimately cell death is implicated in stroke, traumatic brain and spinal cord injury, Huntington's disease, Parkinson's disease (PD), Alzheimer's disease (AD), amyotrophic lateral sclerosis, and other neuropathologies (3, 4, 5, 6, 7, 8).

Disrupted mitochondrial calcium signaling results from either elevated calcium entry or impeded calcium extrusion. In excitable cells such as neurons, these functions are controlled by the mitochondrial calcium uniporter (MCU), a channel that is powered by the steep ΔΨ_m_ and takes up calcium ions into the inner mitochondrial matrix, followed by the mitochondrial solute carrier family 8 sodium/calcium/lithium exchanger, member B1 (NCLX), which acts at a slower and limiting rate to remove calcium ions (9, 10, 11, 12). By modulating neuronal MCU expression, we and others have previously shown that MCU—and therewith mitochondrial calcium uptake—tunes neuronal toxicity in that its reduced expression mitigates the effects of excitotoxic stimuli, whereas its overexpression suffices to cause neuronal death (13, 14, 15, 16, 17). Accordingly, corrections of dysregulated MCU expression or function in models of neurodegenerative diseases such as AD and PD have proven effective for reducing neuronal loss and improving phenotypic outcomes (18, 19, 20, 21). In a similar vein, recent studies aimed at understanding the pathomechanisms of neurodegenerative disorders, including our own investigations on the role of phosphatase and tensin homolog–induced putative kinase 1 or leucine-rich repeat kinase 2 in PD, have demonstrated that dysregulated NCLX function or expression may be a major contributor to the pathophysiology and neuronal demise encountered in these diseases (22, 23, 24, 25, 26).

In this study, we aimed to improve our understanding of the role NCLX plays in neurodegeneration by investigating its contribution to neuronal health under both excitotoxic conditions and during ongoing synaptic activity. To these ends, we used an RNA interference approach to achieve the acute and molecularly selective knockdown of NCLX in primary hippocampal cultures and the dorsal hippocampus of mice. We found that reduced NCLX expression rendered neurons and neuronal mitochondria vulnerable not only to excitotoxic *N*-methyl-d-aspartate (NMDA) stimulation but also to stimuli that trigger synaptic activity and otherwise promote neuronal survival. Moreover, we discovered that NCLX knockdown led to astrodegeneration that—as was the case for neurons—could be seen both *in vitro* and in the cornu ammonis 1 (CA1) region of the hippocampus *in vivo*. Our results thus identify intact NCLX expression and function as a key determinant of neuronal and astroglial fate subsequent to NMDAR-mediated and synaptically triggered intracellular calcium rises and point to NCLX as a potentially highly valuable target for the prevention of cell death in excitotoxic and neurodegenerative disease states.

## Results

### NCLX-directed shRNA effectively reduces NCLX expression in primary hippocampal cultures

**Figure 1:**
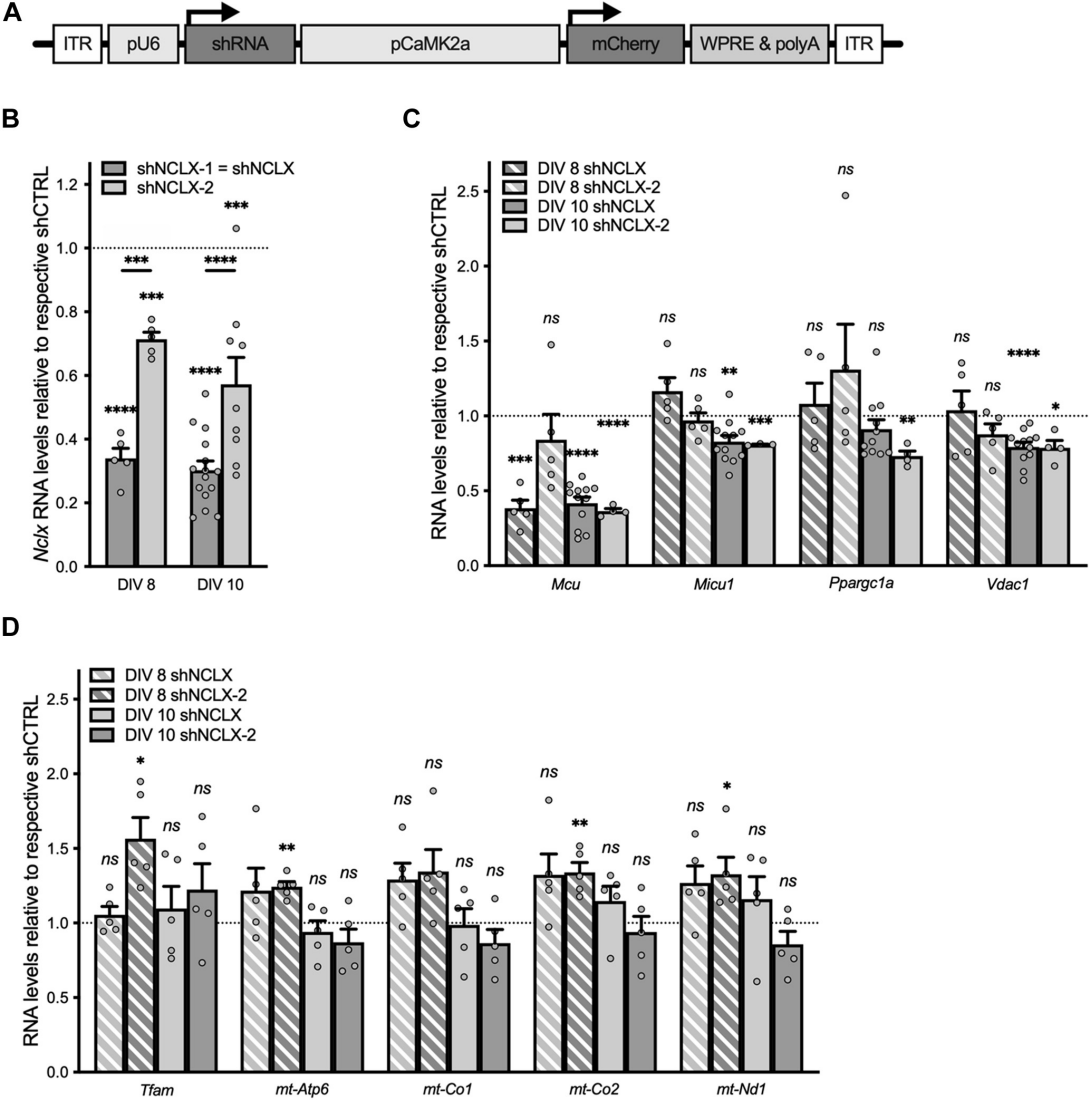
**shRNA-mediated knockdown of NCLX in primary hippocampal cultures.***A,* rAAV construct design. Primary hippocampal cultures were infected on DIV 3 with rAAVs driving expression of two different shRNAs directed against *Nclx* (shNCLX-1 and shNCLX-2; shNCLX-1 is hereafter referred to as shNCLX) or a control shRNA with no known targets in the mouse genome (shCTRL) under control of the U6 promoter (pU6), with mCherry as a marker protein expressed under control of the CaMK2a promoter (pCaMK2a). *B,* quantitative RT–PCR (qRT–PCR) analysis of the *Nclx* message (normalized to *Gusb* and expressed as a fraction of the levels in rAAV-shCTRL-infected cultures) on 5 and 7 days after infection, on DIV 8 and DIV 10 (n = 3–15 independent cultures; two-tailed one-sample *t* tests *versus* a hypothetical value of one; DIV 8: shNCLX-1: *t*_(4)_ = 20.80, *p* < 0.0001, shNCLX-2: *t*_(4)_ = 12.77, *p* = 0.0002. DIV 10: shNCLX-1: *t*_(14)_ = 23.937, *p* < 0.0001, shNCLX-2: *t*_(8)_ = 5.07, *p* = 0.0010; mixed-effects model one-way ANOVA followed by Šidák's multiple comparisons test; shNCLX *versus* shNCLX-2: DIV 8 *t*_(5,5)_ = 5.717, *p* = 0.0001, DIV 10: *t*_(15,9)_ = 5.762, *p* < 0.0001). *C,* qRT–PCR analysis of two genes involved in mitochondrial calcium signaling (*Mcu* and *Micu1*), the major regulator of mitochondrial biogenesis (*Ppargc1a*), and *Vdac1* (all normalized to *Gusb* and expressed as a fraction of the levels observed in rAAV-shCTRL-infected sister cultures) 5 and 7 days after infection, on DIV 8 and DIV 10 (n = 3–12 independent cultures; two-tailed one-sample *t* tests *versus* a hypothetical value of one; *Mcu*: DIV 8 shNCLX: *t*_(4)_ = 11.39, *p* = 0.0003, DIV 8 shNCLX-2: *t*_(4)_ = 0.9359, *p* = 0.4023, DIV 10 shNCLX: *t*_(11)_ = 14.10, *p* < 0.0001, DIV 10 shNCLX-2: *t*_(3)_ = 38.43, *p* < 0.0001; *Micu1*: DIV 8 shNCLX: *t*_(4)_ = 1.846, *p* = 0.1387, DIV 8 shNCLX-2: *t*_(4)_ = 0.5948, *p* = 0.5840, DIV 10 shNCLX: *t*_(11)_ = 4.157, *p* = 0.0016, DIV 10 shNCLX-2: *t*_(3)_ = 4.549, *p* = 0.0199; *Ppargc1a*: DIV 8 shNCLX: *t*_(4)_ = 0.5793, *p* = 0.5934, DIV 8 shNCLX-2: *t*_(10)_ = 1.021, *p* = 0.3651, DIV 10 shNCLX: *t*_(1)_ = 1.397, *p* = 0.1926, DIV 10 shNCLX-2: *t*_(3)_ = 8.033, *p* = 0.0040; *Vdac1*: DIV 8 shNCLX: *t*_(4)_ = 0.2977, *p* = 0.7807, DIV 8 shNCLX-2: *t*_(4)_ = 1.752, *p* = 0.1547, DIV 10 shNCLX: *t*_(11)_ = 6.436, *p* < 0.0001, DIV 10 shNCLX-2: *t*_(3)_ = 4.286, *p* = 0.0233). *D,* qRT–PCR analysis of the mitochondrial transcription factor *Tfam* and four mitochondrial genes that encode members of the electron transport chain (n = 5 independent cultures; two-tailed one-sample *t* tests *versus* a hypothetical value of one; *Tfam*: DIV 8 shNCLX: *t*_(4)_ = 0.9556, *p* = 0.3934, DIV 8 shNCLX-2: *t*_(4)_ = 3.976, *p* = 0.0165, DIV 10 shNCLX: *t*_(4)_ = 0.6539, *p* = 0.5489, DIV 10 shNCLX-2: *t*_(4)_ = 1.286, *p* = 0.2679; *mt-Atp6*: DIV 8 shNCLX: *t*_(4)_ = 1.442, *p* = 0.2227, DIV 8 shNCLX-2: *t*_(4)_ = 7.184, *p* = 0.0020, DIV 10 shNCLX: *t*_(4)_ = 0.7996, *p* = 0.4687, DIV 10 shNCLX-2: *t*_(4)_ = 1.463, *p* = 0.2173; *mt-Co1*: DIV 8 shNCLX: *t*_(4)_ = 2.638, *p* = 0.0577, DIV 8 shNCLX-2: *t*_(4)_ = 2.340, *p* = 0.0794, DIV 10 shNCLX: *t*_(4)_ = 0.1198, *p* = 0.9104, DIV 10 shNCLX-2: *t*_(4)_ = 1.480, *p* = 0.2131; *mt-Co2*: DIV 8 shNCLX: *t*_(4)_ = 2.328, *p* = 0.0804, DIV 8 shNCLX-2: *t*_(4)_ = 5.174, *p* = 0.0066, DIV 10 shNCLX: *t*_(4)_ = 1.491, *p* = 0.2102, DIV 10 shNCLX-2: *t*_(4)_ = 0.5774, *p* = 0.5946; *mt-Nd1*: DIV 8 shNCLX: *t*_(4)_ = 2.349, *p* = 0.0786, DIV 8 shNCLX-2: *t*_(4)_ = 2.848, *p* = 0.0465, DIV 10 shNCLX: *t*_(4)_ = 1.063, *p* = 0.3477, DIV 10 shNCLX-2: *t*_(4)_ = 1.656, *p* = 0.1730). ∗*p* < 0.05, ∗∗*p* < 0.01, ∗∗∗*p* < 0.001, and ∗∗∗∗*p* < 0.0001. Bar graphs show the mean + SEM. DIV, day *in vitro*; ITR, inverted terminal repeat; *Mcu*, mitochondrial uniporter; *Micu1*, mitochondrial calcium uptake 1; *mt-Atp6*, ATP synthase F0 subunit 6, mitochondrial; *mt-Co1*, cytochrome c oxidase I, mitochondrial; *mt-Co2*, cytochrone c oxidase II, mitochondrial; *mt-Nd1*, NADH dehydrtogenase I, mitochondrial; NCLX, solute carrier family 8 sodium/calcium/lithium exchanger, member B1; ns, not significant; pCaMK2a, calcium/calmodulin-dependent protein kinase II alpha promoter; *Ppargc1a*, peroxisome proliferative activated receptor, gamma, coactivator 1 alpha; pU6, U6 small nuclear RNA promoter; qRT–PCR, quantitative reverse transcription polymerase chain reaction; rAAV, recombinant adeno-associated viral vector; shRNA, short hairpin RNA; *Tfam*, transcription factor A, mitochondrial; *Vdac1*, voltage-dependent anion channel 1; WPRE, woodchuck hepatitis virus posttranscriptional regulatory element.

Our principal aim in this study was to explore the possibility that dysregulated NCLX expression plays a pivotal role in excitotoxic cell death within the central nervous system. To test this hypothesis, we employed recombinant adeno-associated viral vectors (rAAVs) to drive the expression of short hairpin RNA (shRNA) directed against the NCLX message in primary hippocampal cultures. More specifically, we designed shRNA sequences against mouse *Nclx* (shNCLX-1, also referred to as shNCLX, and shNCLX-2) and cloned these behind the U6 small nuclear RNA promoter of an rAAV expression cassette that also drives expression of the red fluorescent protein mCherry in excitatory neurons *via* the calcium/calmodulin-dependent protein kinase II alpha (CaMK2a) promoter as an infection marker (Fig. 1*A*). Quantitative reverse transcription polymerase chain reaction (qRT–PCR) analysis revealed that, compared with a control shRNA sequence with no known targets in the mouse genome (shCTRL) (27), rAAV-mediated expression of NCLX-directed shRNA effectively reduced *Nclx* RNA levels in primary hippocampal cultures starting as early as 5 days after infection, on day *in vitro* (DIV) 8 (Fig. 1*B*). Notably, shNCLX (heretofore referred to as shNCLX-1), which targets the mouse NCLX 3′ untranslated region (3′UTR), produced a significantly more efficient knockdown than did shNCLX-2, which targets the NCLX coding sequence (Fig. 1*B*). qRT–PCR analysis of *mCherry* RNA levels suggests that this difference is not a consequence of a higher infection efficiency for rAAV-shNCLX compared with rAAV-shNCLX-2 (Fig. S1*A*).

As attempts to validate NCLX knockdown on the protein level using several commercially available antibodies yielded variable results—consistent with a number of recent studies citing the inadequacy of most anti-NCLX antibodies to detect native NCLX protein (28, 29, 30)—we verified the effectiveness of our shRNAs for reducing NCLX protein expression by cotransfecting human embryonic kidney 293 (HEK293) cells with a plasmid driving the coexpression of enhanced GFP (EGFP) and murine NCLX (pAAV-EGFP.T2A.NCLX; Fig. S2*A*) and plasmids driving the expression of shCTRL, shNCLX, shNCLX-2, or none of these (Fig. S2, *B–D*). Detection using quantitative PCR of pAAV-EGFP.T2A.NCLX, pAAV-shCTRL, pAAV-shNCLX, and pAAV-shNCLX-2 plasmid DNA in lysates from transfected HEK293 cells was used to confirm equal transfection efficiency between samples. As anticipated, both shRNAs targeting the NCLX message reduced heterologous protein expression. Moreover, consistent with our observations regarding their relative efficacy in reducing *Nclx* RNA levels in primary hippocampal cultures (Fig. 1*B*), shNCLX produced a markedly better knockdown than did shNCLX-2 (Fig. S2, *B–D*). It is on account of the higher knockdown efficiency of shNCLX compared with shNCLX-2 that—excepting for the experiments represented in Figs. 1, 4, *A* and *B*, S1, and S2 (see later)—most subsequent analyses were performed using shNCLX in order to best tease out the consequences of disrupted NCLX expression for neuroglial network viability.

We also evaluated—in primary cultures infected with rAAV-shNCLX or rAAV-shNCLX-2 compared with sister cultures infected with rAAV-shCTRL—the expression levels of *Mcu* and mitochondrial calcium uptake 1 (*Micu1*), which control mitochondrial calcium import (10); peroxisome proliferative activated receptor, gamma, coactivator 1 alpha (*Ppargc1a*), a transcriptional coactivator considered to be the master regulator of mitochondrial biogenesis (31, 32, 33); voltage-dependent anion channel 1 (*Vdac1*), which encodes for a voltage-dependent anion channel that facilitates ion and metabolite exchange across the outer mitochondrial membrane (34, 35, 36); transcription factor A, mitochondrial (*Tfam*), a mitochondrial transcription factor that also regulates mitochondrial DNA compaction (33, 37); and a number of mitochondrially encoded components of the electron transport chain with potentially altered expression in neurodegenerative disease: ATP synthase F0 subunit 6, mitochondrial (*mt-Atp6*), cytochrome c oxidase I, mitochondrial (*mt-Co1*), cytochrome c oxidase II, mitochondrial (*mt-Co2*), and NADH dehydrogenase I, mitochondrial (*mt-Nd1*) (31, 38). *Mcu*, *Micu1*, and *Vdac1* were all significantly downregulated for both shRNAs 7 days after infection, on DIV 10 (Fig. 1*C*), suggesting that their altered expression was due to a homeostatic mechanism rather than an off-target effect of either shRNA. In contrast, expression of *Ppargc1a* was not consistently altered in cultures infected with NCLX-targeted shRNA (Fig. 1*C*). Neither *Tfam* nor any of the mitochondrially encoded genes analyzed exhibited altered expression levels in samples infected with rAAV-shNCLX or rAAV-shNCLX-2 7 days after infection, on DIV 10 (Fig. 1*D*). In sum, these data show that the acute disruption of NCLX expression using shRNA is both efficient and likely to be associated with deficient mitochondrial calcium signaling but is not expected to cause a gross disruption of mitochondrial biogenesis or oxidative phosphorylation.

### Reduced NCLX levels impair mitochondrial calcium signaling, alter the mitochondrial glutathione redox potential, and exacerbate mitochondrial membrane potential breakdown during excitotoxic challenge

**Figure 2:**
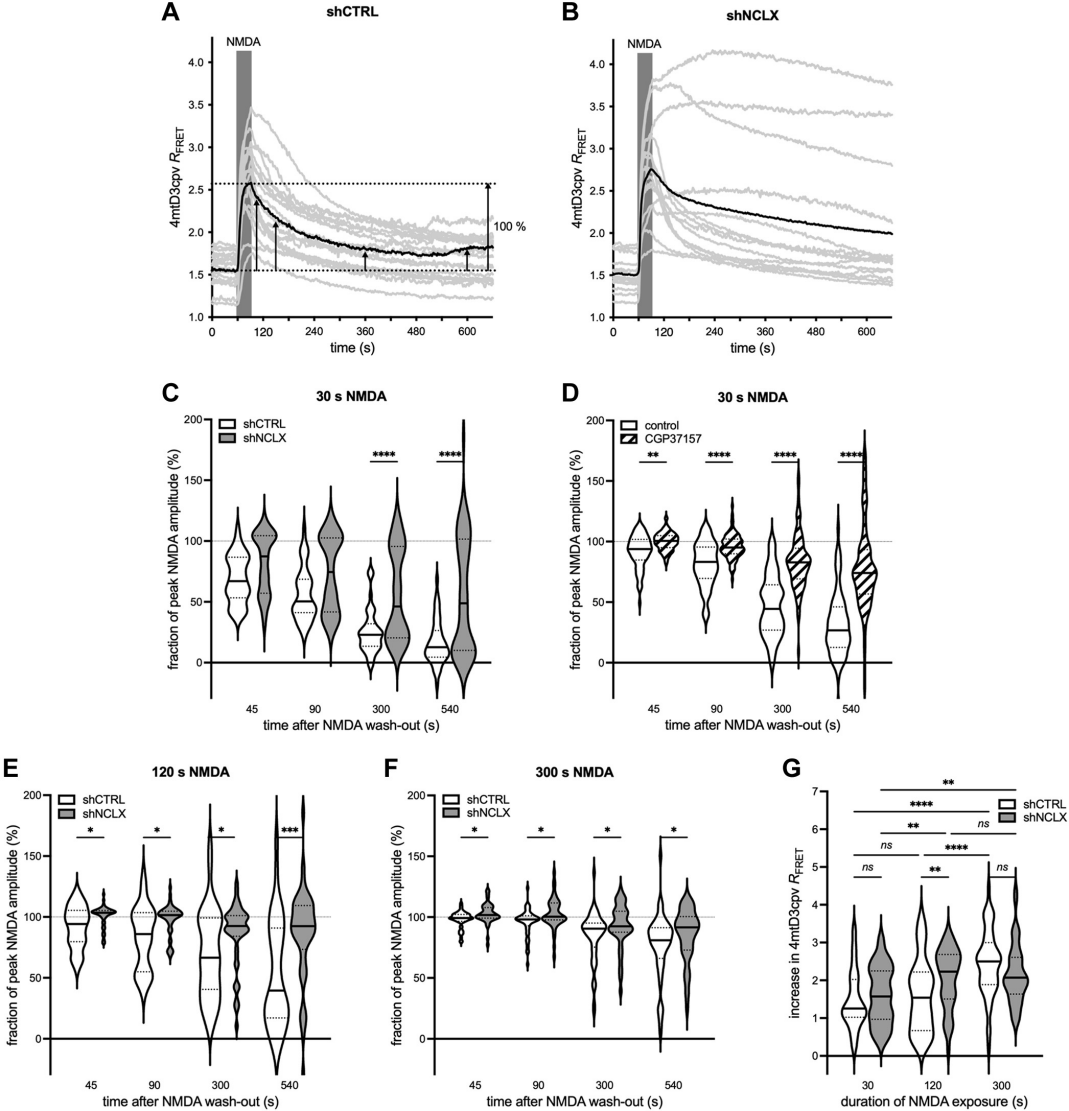
**shRNA-mediated knockdown of NCLX impairs mitochondrial calcium extrusion.***A–C,* NMDA (20 μM) was applied for 30 s to primary hippocampal cultures coinfected with rAAVs driving expression of the mitochondrially targeted FRET-based calcium indicator 4mtD3cpv under control of the CaMK2a promoter and rAAVs driving the expression of either control shRNA (shCTRL) or shRNA directed against NCLX (shNCLX) (shCTRL, n = 46 cells from four coverslips and three independent preparations; shNCLX, n = 56 cells from five coverslips and three independent preparations). *A,* representative mitochondrial calcium responses, quantified using the crosstalk- and bleaching-corrected 4mtD3cpv FRET ratio, *R*_FRET_, to 30 s NMDA stimulation in rAAV-shCTRL-infected neurons from a single coverslip. In subsequent analyses, amplitudes of mitochondrial calcium levels were measured at the time points indicated by the *arrows* (45, 90, 300, and 540 s after NMDA washout) and normalized to the peak NMDA response for each cell (*gray*, individual cells; *black*, their mean). *B,* representative mitochondrial calcium responses to 30 s NMDA stimulation in rAAV-shNCLX-infected neurons from a single coverslip (*gray*, individual cells; *black*, their mean). *C,* quantification of the decay of 30 s NMDA-evoked mitochondrial calcium rises 45, 90, 300, and 540 s after NMDA washout (normalized to the peak NMDA response) (shCTRL: n = 46 cells from four coverslips and three independent preparations; shNCLX: n = 56 cells from five coverslips and three independent preparations; Kruskal–Wallis test followed by Dunn's multiple comparisons test; shNCLX *versus* shCTRL: 45 s *Z*_(56,46)_ = 1.831, *p* = 0.2683, 90 s *Z*_(56,46)_ = 2.130, *p* = 0.1328, 300 s *Z*_(56,46)_ = 4.281, *p* < 0.0001, 540 s *Z*_(56,46)_ = 5.173, *p* < 0.0001). *D,* NMDA (20 μM) was applied for 30 s to primary hippocampal cultures infected with rAAVs driving the expression of 4mtD3cpv under control of the CaMK2a promoter in the presence (or not) of the NCLX inhibitor CGP37157 (10 mM). When used, CGP37157 was present in the culture medium starting ≥5 min prior to and during the entire course of the experiment. Shown are the amplitudes of NMDA-evoked responses (normalized to the peak response) at 45, 90, 300, and 540 s after NMDA washout (vehicle n = 76 cells from three coverslips and three independent preparations, CGP37157 n = 93 cells from three coverslips and three independent preparations; Kruskal–Wallis test followed by Dunn's multiple comparisons test; CGP37157 *versus* vehicle: 45 s *Z*_(76,93)_ = 2.840, *p* = 0.0045, 90 s *Z*_(76,93)_ = 4.150, *p* < 0.0001, 300 s *Z*_(76,93)_ = 6.441, *p* < 0.0001, and 540 s *Z*_(76,93)_ = 6.872, *p* < 0.0001). *E* and *F,* quantification of the decay of NMDA-evoked mitochondrial calcium rises in rAAV-shCTRL and rAAV-shNCLX 45, 90, 300, and 540 s after NMDA washout (normalized to the peak NMDA response) for stimuli lasting 120 s (*E*; shCTRL n = 35 cells from three coverslips and three independent preparations, shNCLX n = 48 cells from four coverslips and three independent preparations; Kruskal–Wallis test followed by Dunn's multiple comparisons test; shNCLX *versus* shCTRL: 45 s *Z*_(35,48)_ = 2.120, *p* = 0.0340, 90 s *Z*_(35,48)_ = 2.494, *p* = 0.0126, 300 s *Z*_(35,48)_ = 1.960, *p* = 0.0500, 540 s *t*_(35,48)_ = 3.687, *p* = 0.0002) and 300 s (*F*; shCTRL n = 52 cells from five coverslips and three independent preparations, shNCLX n = 45 cells from four coverslips and three independent preparations; Kruskal–Wallis test followed by Dunn's multiple comparisons test; shNCLX *versus* shCTRL: 45 s *Z*_(52,45)_ = 2.069, *p* = 0.0385, 90 s *Z*_(52,45)_ = 2.402, *p* = 0.0163, 300 s *Z*_(52,45)_ = 2.487, *p* = 0.0129, 540 s *Z*_(52,45)_ = 2.263, *p* = 0.0237). *G,* quantification of the peak amplitude of NMDA-evoked calcium rises in rAAV-shCTRL-infected and rAAV-shNCLX-infected cells stimulated with NMDA for 30, 120, or 300 s as in *C*, *F*, and *G* (Kruskal–Wallis test followed by Dunn's multiple comparisons test; shNCLX *versus* shCTRL: 30 s *Z*_(46,56)_ = 0.9176, *p* = 0.3588, 120 s *Z*_(35,48)_ = 2.598, *p* = 0.0094, 300 s *Z*_(52,45)_ = 1.640, *p* = 0.1011; shCTRL: 30 s *versus* 120 s *Z*_(46,35)_ = 0.7152, *p* = 0.4745, 30 s *versus* 300 s *Z*_(46,52)_ = 5.418, *p* < 0.0001, 120 s *versus* 300 s *Z*_(35,52)_ = 4.282, *p* < 0.0001; shNCLX: 30 s *versus* 120 s *Z*_(56,48)_ = 2.822, *p* = 0.0048, 30 s *versus* 300 s *Z*_(56,45)_ = 2.898, *p* = 0.0038, 120 s *versus* 300 s *Z*_(48,45)_ = 0.1205, *p* = 0.9041). ns, ∗*p* < 0.05, ∗∗*p* < 0.01, ∗∗∗*p* < 0.001, ∗∗∗∗*p* < 0.0001. Bar graphs show the mean + SEM. Violin plots show the probability density of the data as well as median and quartile divisions. CaMK2a, calcium/calmodulin dependent protein kinase II alpha; FRET, Förster resonance energy transfer; NCLX, solute carrier family 8 sodium/calcium/lithium exchanger, member B1; NMDA, *N*-methyl-d-aspartate; ns, not significant; rAAV, recombinant adeno-associated viral vector.

NCLX is thought to be the primary mediator of mitochondrial calcium extrusion in excitable cells (39, 40). We therefore reasoned that its knockdown in neurons would impair their recovery from stimulus-triggered mitochondrial calcium rises such as those evoked by brief pharmacological stimulation with NMDA, an *in vitro* model for excitotoxicity (41). To test this hypothesis, we performed mitochondrial calcium imaging using the Förster resonance energy transfer (FRET)-based genetically encoded mitochondrial calcium indicator 4mtD3cpv (42) in excitatory neurons coinfected with the aforedescribed shRNA constructs. Coexpression of shCTRL or shNCLX was reliably determined using mCherry fluorescence, and only mCherry^+^ cells were analyzed. Notably, both mCherry and 4mtD3cpv exhibited marked cell-to-cell variability in their basal fluorescence intensity (Fig. S1*B*). These differences are suggestive of an inhomogeneity in the efficiency of infection between individual neurons that might also result in disparate degrees of NCLX knockdown. Consistent with this idea, and with an important role for NCLX in neuronal mitochondrial calcium homeostasis, NCLX knockdown impaired mitochondrial calcium extrusion, quantified using the cross-talk- and bleaching-corrected FRET ratio (*R*_FRET_), following a brief (30 s) NMDA stimulation, but only in a subset of cells (Fig. 2, *A–C*). This inhibition of mitochondrial calcium clearance was similar to that mediated by the pharmacological NCLX antagonist, CGP37157 (Fig. 2*D*). Notably, CGP37157 treatment was associated with a marked reduction in the amplitude of calcium rises (vehicle: increase in *R*_FRET_ = 0.94 ± 0.06; CGP37157: increase in *R*_FRET_ = 0.58 ± 0.03; two-tailed independent-samples Mann–Whitney test; *U*_(76,93)_ = 1884, *p* < 0.0001), probably because of an unspecific inhibitory effect of CGP37157, for instance on L-type voltage-gated calcium channels (43). NCLX knockdown also inhibited mitochondrial calcium clearance following more robust excitotoxic stimuli lasting 120 s (Fig. 2*E*) and 300 s (Fig. 2*F*). In these cases, however, the population of rAAV-shNCLX-infected cells exhibited a more uniform disruption of mitochondrial calcium recovery. NMDA stimuli lasting 120 s, but not 30 s or 300 s stimulus, evoked larger amplitude mitochondrial calcium rises in rAAV-shNCLX-infected cells than in rAAV-shCTRL-infected cells (Fig. 2*G*). Moreover, as expected, longer-lasting stimuli were associated with larger amplitude mitochondrial calcium rises (Fig. 2*G*). In sum, these results indicate that NCLX knockdown results in a functional inhibition of mitochondrial calcium recovery.

**Figure 3:**
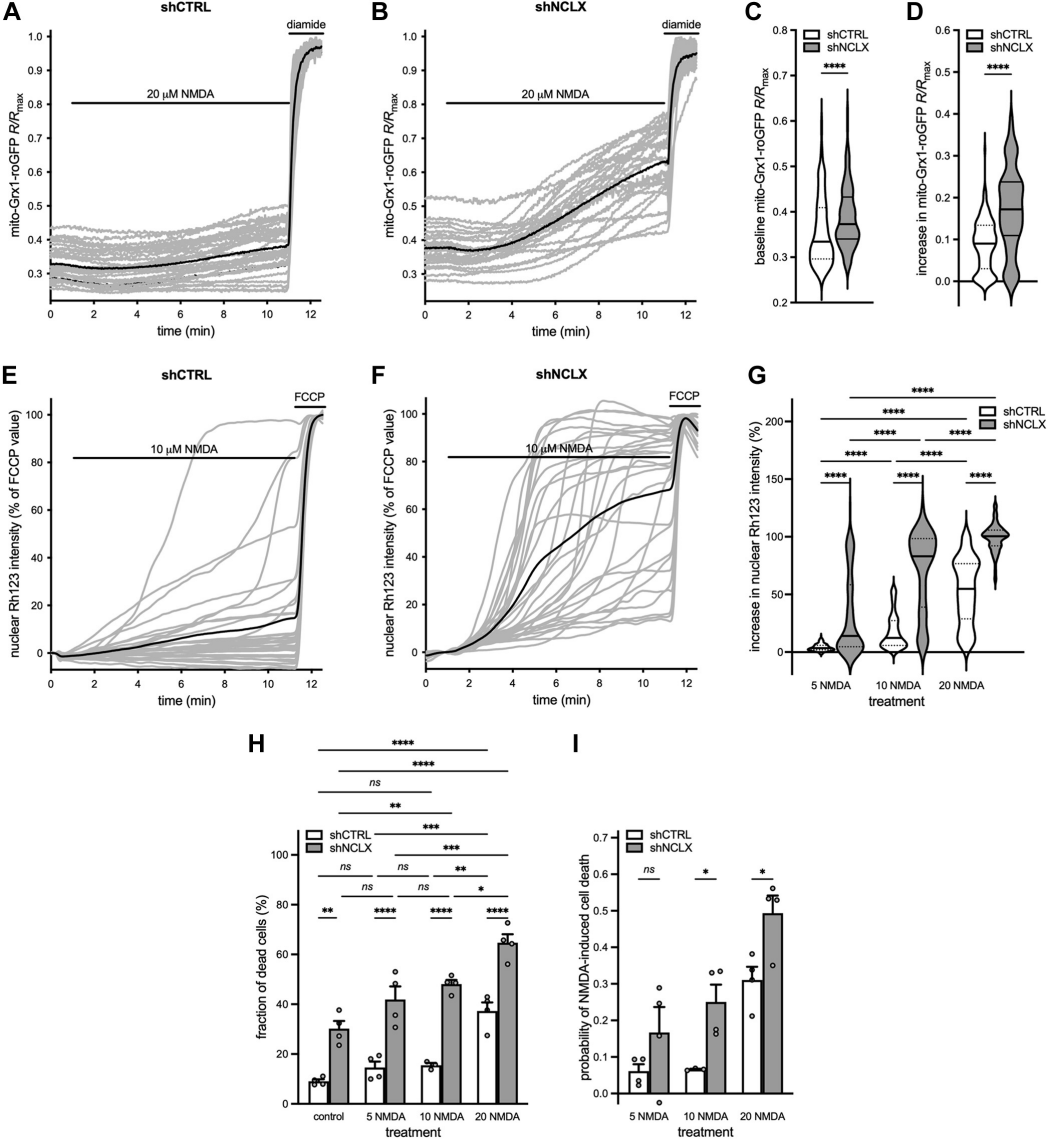
**shRNA-mediated knockdown of NCLX alters the mitochondrial redox state, sensitizes ΔΨ**_**m**_**to breakdown, and renders cells more vulnerable to excitotoxic stimuli.***A–D,* NMDA (20 μM) was applied to primary hippocampal cultures coinfected with rAAVs driving the expression of mito-Grx1-roGFP2 and either shCTRL or shNCLX. Complete oxidation of the sensor was achieved with 0.5 mM diamide (shCTRL: n = 171 cells from eight coverslips and three independent preparations; shNCLX: n = 119 cells from eight coverslips and three independent preparations). *A* and *B,* representative levels of the mito-Grx1-roGFP 405/480 ratio, *R* (expressed as a fraction of the maximum ratio observed during diamide treatment, *R*_max_) prior to and during NMDA application in rAAV-shCTRL-infected (*A*) and rAAV-shNCLX-infected neurons (*B*) on a single coverslip each (*gray*, individual cells; *black*, their mean). *C,* quantification of baseline *R*/*R*_max_ in rAAV-shCTRL-infected and rAAV-shNCLX-infected neurons measured in the last 10 s prior to NMDA (two-tailed independent-samples Mann–Whitney test; *U*_(171,119)_ = 7265, *p* < 0.0001). *D,* changes in mitochondrial redox state quantified as the amplitude of the baseline-subtracted *R*/*R*_max_ ratio following 10 min of NMDA treatment (two-tailed independent-samples Mann–Whitney test; *U*_(171,119)_ = 4905, *p* < 0.0001). *E–G,* NMDA (5, 10, or 20 μM) was applied to primary hippocampal cultures loaded with Rh123 and infected with rAAVs driving the expression of either shCTRL or shNCLX. The mitochondrial uncoupler FCCP (5 μM) was used to trigger complete ΔΨ_m_ breakdown (5 μM NMDA: shCTRL n = 185 cells from seven coverslips and five independent preparations, shNCLX n = 103 cells from five coverslips and four independent preparations; 10 μM NMDA: shCTRL n = 133 cells from five coverslips and four independent preparations, shNCLX n = 122 cells from five coverslips and four independent preparations; 20 μM NMDA: shCTRL n = 160 cells from four coverslips and four independent preparations, and shNCLX n = 91 cells from four coverslips and four independent preparations). *E* and *F,* representative nuclear Rh123 fluorescence (expressed as a percent of the FCCP-triggered fluorescence maximum, with baseline set to 0%) during 10 μM NMDA treatment in rAAV-shCTRL-infected (*E*) and rAAV-shNCLX-infected neurons (*F*) on a single coverslip each (*gray*, individual cells; *black*, their mean). *G,* quantification of ΔΨ_m_ loss as the peak amplitude during the first 10 min of NMDA treatment (Kruskal–Wallis test followed by Dunn's multiple comparisons test; shCTRL *versus* shNCLX: 5 μM *Z*_(185,103)_ = 7.295, *p* < 0.0001, 10 μM *Z*_(133,122)_ = 8.977, *p* < 0.0001, 20 μM *Z*_(160,91)_ = 7.229, *p* < 0.0001; shCTRL: 5 *versus* 10 μM *Z*_(185,133)_ = 6.452, *p* < 0.0001, 5 *versus* 20 μM *Z*_(185,160)_ = 14.01, *p* < 0.0001, 10 *versus* 20 μM *Z*_(133,160)_ = 6.639, *p* < 0.0001; shNCLX: 5 *versu*s 10 μM *Z*_(103,122)_ = 7.188, *p* < 0.0001, 5 *versus* 20 μM *Z*_(103,91)_ = 10.88, *p* < 0.0001, 10 *versus* 20 μM *Z*_(122,91)_ = 4.532; *p* < 0.0001). *H* and *I,* NMDA (0, 5, 10, or 20 μM; 10 min) was applied to primary hippocampal cultures, and the numbers of live and dead cells assessed 16 to 24 h later (n = 3–4 independent cultures). *H,* proportions of dead cells (ordinary two-way ANOVA followed by Tukey's multiple comparisons test; main effect of shRNA *F*_(1,23)_ = 154.3, *p* < 0.0001; shCTRL *versus* shNCLX: control *q*_(4,4)_ = 6.993, *p* = 0.0012, 5 μM *q*_(4,4)_ = 9.031, *p* < 0.0001; 10 μM NMDA *q*_(4,3)_ = 9.979, *p* < 0.0001; 20 μM *q*_(4,4)_ = 9.061, *p* < 0.0001; main effect of NMDA concentration *F*_(3,23)_ = 38.32, *p* < 0.0001; shCTRL: control *versus* 5 μM *q*_(4,4)_ = 1.826, *p* = 0.8930, control *versus* 10 μM *q*_(4,3)_ = 1.966, *p* = 0.8526, control *versus* 20 μM *q*_(4,4)_ = 9.333, *p* < 0.0001, 5 *versus* 10 μM *q*_(4,3)_ = 0.2751, *p* > 0.9999, 5 *versus* 20 μM *q*_(4,4)_ = 7.507, *p* = 0.0005, 10 *versus* 20 μM *q*_(3,4)_ = 6.675, *p* = 0.0020; shNCLX: control *versus* 5 μM *q*_(4,4)_ = 3.865, *p* = 0.1626, control *versus* 10 μM *q*_(4,4)_ = 5.909, *p* = 0.0073, control *versus* 20 μM *q*_(4,4)_ = 11.40, *p* < 0.0001, 5 *versus* 10 μM *q*_(4,4)_ = 2.044, *p* = 0.8269, 5 *versus* 20 μM *q*_(4,4)_ = 7.537, *p* = 0.0005, 10 *versus* 20 μM *q*_(4,4)_ = 5.493, *p* = 0.0145). *I,* quantification of the probability of cells dying specifically because of NMDA treatment, which takes into account elevated levels of basal cell death (see the Experimental procedures section; ordinary two-way ANOVA followed by Šídák's multiple comparisons test; main effect of shRNA *F*_(1,17)_ = 18.12, *p* = 0.0005; shCTRL *versus* shNCLX: 5 μM *t*_(4,4)_ = 1.694, *p* = 0.2914; 10 μM *t*_(3,4)_ = 2.735, *p* = 0.0417; 20 μM *t*_(4,4)_ = 2.927, *p* = 0.0279). ns, ∗*p* < 0.05, ∗∗*p* < 0.01, ∗∗∗*p* < 0.001, and ∗∗∗∗*p* < 0.0001. Bar graphs show the mean + SEM. Violin plots show the probability density of the data as well as median and quartile divisions. FCCP, carbonyl cyanide-*p*-trifluoromethoxyphenylhydrazone; NCLX, solute carrier family 8 sodium/calcium/lithium exchanger, member B1; NMDA, *N*-methyl-d-aspartate; ns, not significant; rAAV, recombinant adeno-associated viral vector; Rh123, rhodamine 123; ΔΨ_m_, mitochondrial membrane potential.

Altered cellular oxidation–reduction equilibrium (redox state) and oxidative stress represent early events in mitochondrial dysfunction, and increased reactive oxygen species (ROS) production can trigger neuronal damage and death (44, 45). Excitotoxic stimuli—modeled by the stimulation of NMDARs *via* bath application of NMDA as in this study—have been shown to increase ROS production in cultures of both cerebellar granule cells and forebrain neurons subsequent to excessive mitochondrial calcium influx (46, 47, 48, 49, 50, 51). Previous studies indeed indicate that knockdown of NCLX expression enhances mitochondrial ROS production (52). We therefore reasoned that disruption of mitochondrial calcium clearance following NCLX knockdown would exacerbate mitochondrial redox state disruption in neurons challenged with NMDA. To test this hypothesis, we employed the mitochondrial matrix–targeted ratiometric glutathione redox potential indicator, mito-Grx1-roGFP2 (47, 53), to assess the mitochondrial redox state following NMDA bath application in neurons infected with rAAV-shCTRL or rAAV-shNCLX. In these experiments, the mito-Grx1-roGFP 405/480 ratio, *R* (expressed as a fraction of *R*_max_, the maximum ratio achieved by treatment of cells with the oxidizing reagent diamide) was measured prior to and during NMDA application. Compared with rAAV-shCTRL-infected neurons, rAAV-shNCLX-infected neurons were both more highly oxidized at baseline (Fig. 3, *A–C*) and exhibited a more pronounced NMDA-triggered increase in oxidation (Fig. 3, *A*, *B*, and *D*). These data confirm that intact NCLX expression is important for maintaining mitochondrial redox state under basal conditions and following an excitotoxic challenge.

Another important parameter that describes mitochondrial health and is disturbed during excitotoxic neuronal damage is ΔΨ_m_. Mitochondrial calcium overload is thought to play a key role in the disruption of ΔΨ_m_ that follows an excitotoxic insult (54, 55, 56). Having observed that mitochondrial calcium recovery was functionally impaired for brief (30 s) to prolonged (300 s) excitotoxic NMDA stimulation in neurons expressing shRNA directed against NCLX, we reasoned that these cells may similarly be sensitized not only to severe (20 μM NMDA) but also to milder (5–10 μM NMDA) excitotoxic stimulation, which we had previously observed to trigger sustained mitochondrial calcium transients and cell death (13). To quantify the effect of NCLX knockdown on ΔΨ_m_ breakdown, we employed the ΔΨ_m_ indicator rhodamine 123 (Rh123) in quenching mode (57, 58). Consistent with our expectations, rAAV-shNCLX-infected neurons responded to NMDA treatment with a much more rapid and robust breakdown of ΔΨ_m_ than rAAV-shCTRL-infected neurons, also for relatively mild excitotoxic insults (Fig. 3, *E–G*). The degree of ΔΨ_m_ breakdown was dependent on the intensity of NMDA stimulation (Fig. 3*G*). Taken together, these findings demonstrate that reduced NCLX expression in neurons impairs mitochondrial calcium clearance, disturbs the mitochondrial redox state, and exacerbates ΔΨ_m_ breakdown following an excitotoxic challenge. Moreover, they suggest that disrupted NCLX expression may result in a reduction in the intensity of excitotoxic challenge required to trigger irreparable mitochondrial damage and subsequent death in neurons.

### NCLX knockdown enhances neuronal death at baseline and following excitotoxic challenge

To assess whether NCLX knockdown indeed renders neurons more susceptible to death following milder excitotoxic insults, we examined the levels of cell death in primary cultures infected with rAAV-shCTRL or rAAV-shNCLX and challenged with different concentrations of NMDA. In line with our observations that NCLX knockdown sensitized ΔΨ_m_ to breakdown following a mild excitotoxic stimulus and that the mitochondrial redox state was disturbed even at baseline, we found that vehicle-stimulated rAAV-shNCLX-infected cells (control) and rAAV-shNCLX-infected cells that were stimulated with NMDA (5, 10, or 20 μM) exhibited higher levels of cell death than rAAV-shCTRL-infected cells (Fig. 3*H*). Furthermore, cell death rates were generally greater for higher NMDA concentrations (Fig. 3*H*). Higher levels of cell death in NMDA-treated rAAV-shNCLX-infected cells could be due to higher sensitivity to NMDA or could simply result from higher levels of basal cell death. To distinguish between these possibilities, we calculated the NMDA-dependent probability of a cell dying (see the Experimental procedures section for details). This analysis revealed that rAAV-shNCLX-infected cells are indeed more sensitive to NMDA than rAAV-shCTRL-infected cells (Fig. 3*I*). Thus, consistent with its effect on mitochondrial health, knockdown of NCLX increases neuronal susceptibility to excitotoxic insults.

### NCLX knockdown decreases the viability of neurons and glia

**Figure 4:**
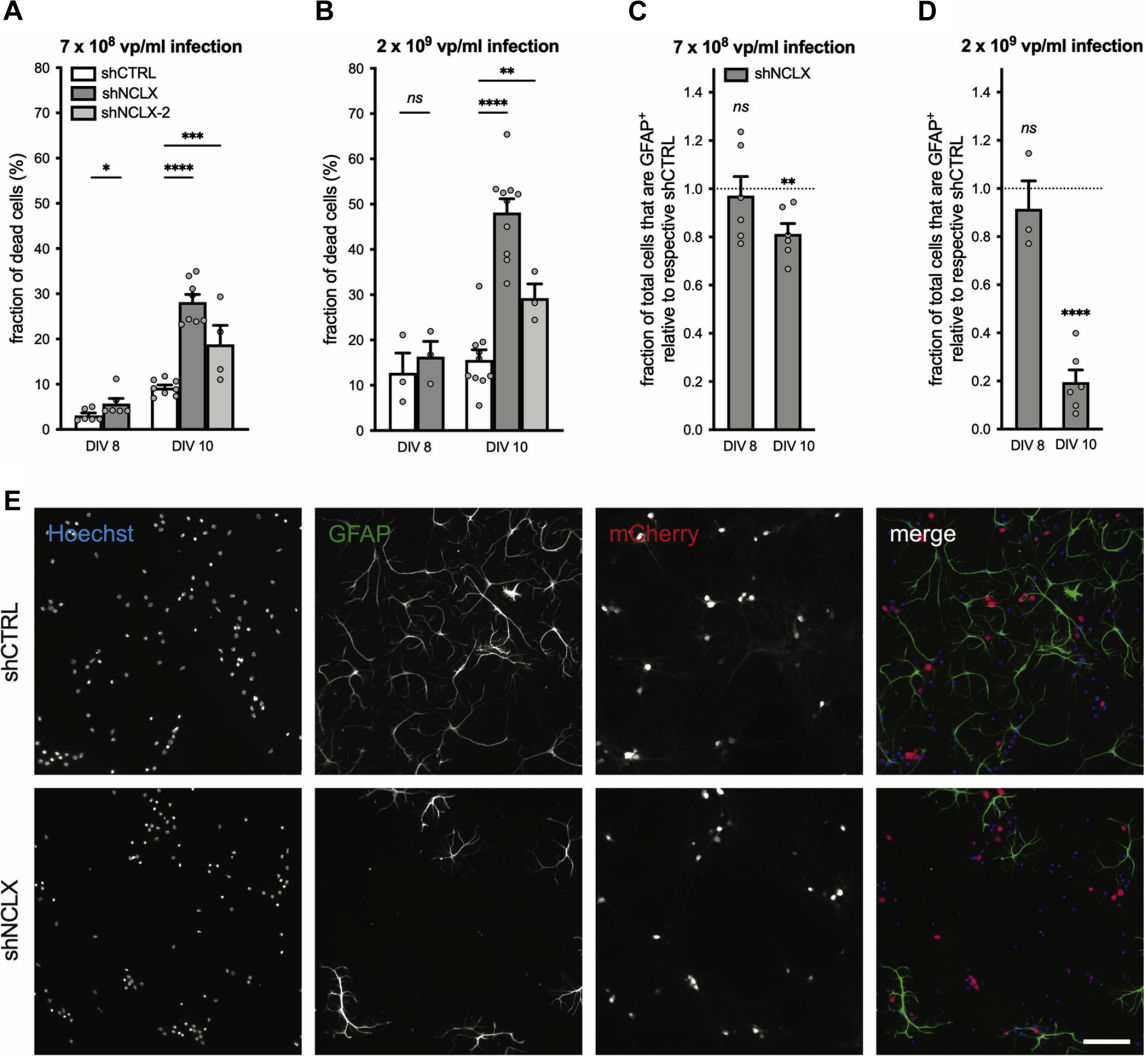
**shRNA-mediated knockdown of NCLX leads to death of neurons and astrocytes *in vitro*.***A–E,* primary hippocampal cultures were infected on DIV 4 with rAAVs driving the expression of either shRNA directed against NCLX (shNCLX-1 = shNCLX or shNCLX-2) or control shRNA (shCTRL), fixed on DIV 8 or DIV 10, and subsequently processed for anti-GFAP immunochemistry. *A* and *B,* quantification of the fraction of total dead and dying cells for cultures infected with 7 × 10^8^ viral particles (vp)/ml (*A*; DIV 8: shCTRL n = 6 independent cultures, shNCLX n = 6 independent cultures; Wilcoxon matched-pairs signed rank test; *p* = 0.0312; DIV 10: shCTRL n = 8 independent cultures, shNCLX n = 8 independent cultures, shNCLX-2 n = 4 independent cultures; mixed-effects model one-way ANOVA followed by Dunnett's multiple comparisons test; *F*_(4,14)_ = 50.45, *p* < 0.0001; shCTRL *versus* shNCLX *q*_(8,8)_ = 13.94, *p* < 0.0001; shCTRL *versus* shNCLX-2 *q*_(8,4)_ = 5.634, *p* = 0.0002) or 2 × 10^9^ vp/ml (*B*; DIV 8: shCTRL n = 3 independent cultures, shNCLX n = 3 independent cultures; two-tailed paired-samples *t* test; *t*_(2)_ = 2.373, *p* = 0.1410; DIV 10: shCTRL n = 10 independent cultures, shNCLX n = 10 independent cultures; shNCLX-2 n = 3 independent cultures; mixed-effects model one-way ANOVA followed by Dunnett's multiple comparisons test; *F*_(2,11)_ = 132.5, *p* < 0.0001; shCTRL *versus* shNCLX *q*_(10,10)_ = 16.25, *p* < 0.0001, shCTRL *versus* shNCLX-2 *q*_(10,3)_ = 4.199, *p* = 0.0029). *C* and *D,* quantification of the fraction of GFAP^+^ cells in rAAV-shNCLX-infected cultures, normalized to values obtained from sister cultures infected with rAAV-shCTRL for an infection rate of 7 × 10^8^ vp/ml (*C*; n = 6 independent culture pairs for all conditions; two-tailed one-sample *t* tests *versus* a hypothetical value of one; DIV 8 *t*_(5)_ = 0.3695, *p* = 0.7269, DIV 10 *t*_(5)_ = 4.326, *p* = 0.0075) or 2 × 10^9^ vp/ml (*D*; n = 3 independent culture pairs for all conditions; two-tailed one-sample *t* tests *versus* a hypothetical value of one; DIV 8 *t*_(2)_ = 0.7262, *p* = 0.5432, DIV 10 *t*_(2)_ = 10.24, *p* = 0.0094). *E,* representative images showing nuclear Hoechst stain (*blue*), anti-GFAP immunocytochemistry (*green*), and mCherry fluorescence (*red*) in shCTRL-infected and rAAV-shNCLX-infected cultures (2 × 10^9^ vp/ml) fixed on DIV 10. The scale bar represents 100 μm. ∗*p* < 0.05, ∗∗*p* < 0.01, ∗∗∗*p* < 0.001, and ∗∗∗∗*p* < 0.0001. Bar graphs show the mean + SEM. DIV, day *in vitro*; GFAP, glial fibrillary acidic protein; NCLX, solute carrier family 8 sodium/calcium/lithium exchanger, member B1; rAAV, recombinant adeno-associated viral vector; shRNA, short hairpin RNA; vp, viral particles.

We next assessed in more detail how decreased NCLX expression impacts cell health in the absence of any excitotoxic stimulus. In these experiments, we used two different viral infection rates in order to gain better insight into how NCLX levels impact cell survival: 7 × 10^8^ viral particles/ml (Fig. 4, *A* and *C*) and a threefold higher infection, 2 × 10^9^ viral particles/ml (Fig. 4, *B* and *D*). This rAAV-shNCLX infection rate was associated with only slightly higher basal cell death rates than rAAV-shCTRL infection on DIV 8, but with dramatically higher basal cell death rates on DIV 10 (Fig. 4, *A* and *E*). Consistent with the idea that cell death is linked to NCLX knockdown rather than a nonspecific effect of rAAV-shNCLX, we also confirmed a statistically significant increase in basal cell death rates for shNCLX-2 at this infection rate on DIV 10 (Fig. 4*A*). Increasing the viral load by three times to 2 × 10^9^ viral particles/ml, which resulted in a significantly greater reduction of *Nclx* RNA on DIV 10 (shNCLX/shCTRL: 7 × 10^8^ particles/ml 0.30 ± 0.11, n = 15, 2 × 10^9^ particles/ml 0.11 ± 0.04, n = 5; shNCLX-2/shCTRL: 7 × 10^8^ particles/ml 0.57 ± 0.25, n = 9, 2 × 10^9^ particles/ml 0.28 ± 0.00, n = 2; ordinary two-way ANOVA followed by Tukey's multiple comparisons test; main effect of infection rate *F*_(1,28)_ = 10.79, *p* = 0.0027; shNCLX/shCTRL *q*_(15,5)_ = 4.646, *p* = 0.0138, shNCLX-2/shCTRL *q*_(9,2)_ = 4.646, *p* = 0.0138; see also Fig. 1*B*), resulted in an even more dramatic loss of cells on DIV 10 for both shNCLX and shNCLX-2 (Fig. 4*B*). Indeed, viral load had a significant influence on overall cell death for rAAV-shNCLX-infected cells on both DIV 8 (Fig. 4, *A* and *B*; ordinary two-way ANOVA followed by Šídák's multiple comparisons test; main effect of viral load *F*_(1,7)_ = 13.24, *p* = 0.0083; 7 × 10^8^ *versus* 2 × 10^9^: shCTRL *t*_(6,3)_ = 3.356, *p* = 0.0094, shNCLX *t*_(6,3)_ = 3.691, *p* = 0.0048) and DIV 10 (Fig. 4, *A* and *B*; mixed-effects model two-way ANOVA followed by Šídák's multiple comparisons test; main effect of viral load *F*_(1,9)_ = 19.68, *p* = 0.0016; 7 × 10^8^ *versus* 2 × 10^9^: shCTRL *t*_(8,10)_ = 2.7081, *p* = 0.0645, shNCLX *t*_(8,10)_ = 6.638, *p* = 0.0002, shNCLX-2 *t*_(4,3)_ = 2.733, *p* = 0.0620). These data indicate that the extent to which NCLX expression is reduced from basal levels is a determinant for its impact on neuronal health.

It has been reported that NCLX is considerably more highly expressed by astrocytes than neurons (59, 60, 61). Moreover, the rAAV expression system we employed (serotype 1/2) is capable of mediating transgene expression in astrocytes (*e.g.*, (62)) and drives the expression of shRNA under the control of a ubiquitous eukaryotic RNA polymerase III U6 promoter. It thus seems plausible that not only neurons but also astrocytes may be affected by rAAV-shNCLX infection in our experimental setup. In addition to quantifying overall cell death rates in cultures infected with NCLX-targeted shRNA (Fig. 4, *A* and *B*), we therefore also quantified viable cells immunopositive for the astrocytic marker protein glial fibrillary acidic protein (GFAP) (Fig. 4, *C* and *D*). In keeping with the idea that NCLX knockdown leads to astrocytic cell death, we found a significantly smaller proportion of viable GFAP^+^ cells on DIV 10 for both our standard infection rate (Fig. 4*C*) and for the three-times greater infection rate (Fig. 4, *D* and *E*) in cultures that had been infected with rAAV-shNCLX compared with those infected with rAAV-shCTRL. Moreover, like the overall basal cell death rate, this effect was greater for the higher infection rate on DIV 10 (DIV 8: two-tailed independent-samples *t* test; *t*_(7)_ = 0.3946, *p* = 0.7049; DIV 10: two-tailed independent-samples *t* test; *t*_(7)_ = 6.813, *p* = 0.0003). The observed reduction in numbers of GFAP^+^ cells in rAAV-shNCLX-infected cultures is unlikely to reflect an impairment of glial cell proliferation, since the proportion of GFAP^+^ cells in sister cultures infected with rAAV-shCTRL either was decreased (7 × 10^8^ viral particles/ml infection: DIV 8 17.74 ± 2.57%; DIV 10 13.70 ± 2.74%; two-tailed paired-samples *t* test; *t*_(5)_ = 3.831, *p* = 0.0122) or was unchanged in this time frame (2 × 10^9^ viral particles/ml infection: DIV 8 22.21 ± 1.85%; DIV 10 22.00 ± 1.62%; two-tailed paired-samples *t* test; *t*_(2)_ = 0.6310, *p* = 0.5926). Thus, NCLX knockdown leads to a dose-dependent loss of both neurons and astrocytes between DIV 8 and DIV 10 in our primary hippocampal cultures.

**Figure 5:**
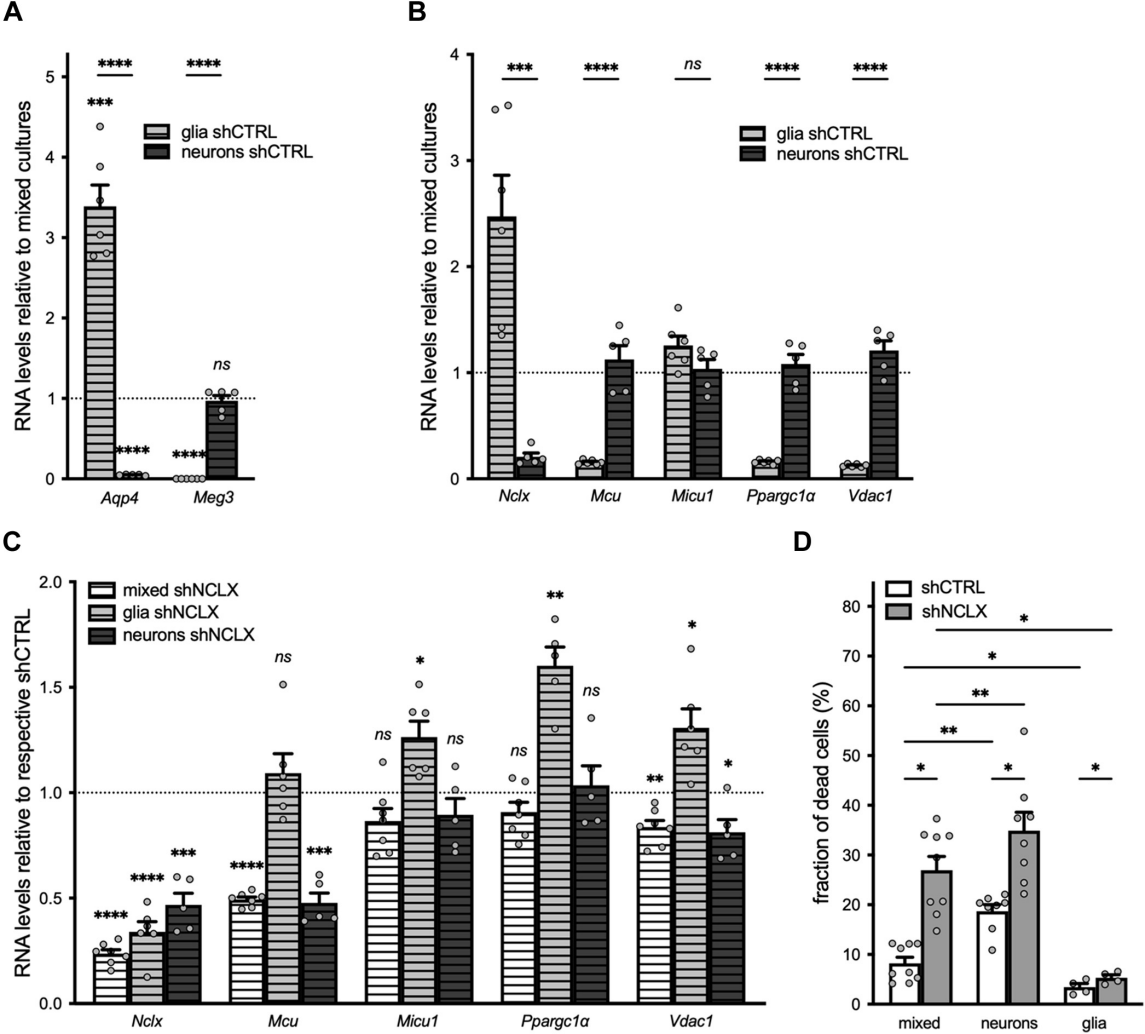
**shRNA-mediated knockdown of NCLX triggers gene expression changes in both neurons and glia.***A* and *B,* neuron-depleted (glia) and glia-depleted (neurons) sister cultures of primary hippocampal cells were cultivated in parallel to mixed cultures containing both neurons and glia, and all were infected with rAAV-shCTRL on DIV 3. RNA was harvested on DIV 10 and processed for qRT–PCR. Expression levels of target genes were normalized to *Gusb*, and then the ratio of glial or neuronal expression to the expression in mixed cultures calculated for each gene (mixed n = 6 independent cultures; glia n = 6 independent cultures; and neurons n = 5 independent cultures). *A,* relative expression levels of the cell type–specific genes *Aqp4* (for astrocytes) and *Meg3* (for neurons) (comparisons to mixed cultures: two-tailed one-sample *t* tests *versus* a hypothetical value of one; *Aqp4*: glia *t*_(5)_ = 9.065, *p* = 0.0003, neurons *t*_(4)_ = 297.2, *p* < 0.0001; *Meg3*: two-tailed one-sample *t* tests *versus* a hypothetical value of one; glia *t*_(5)_ = 13,700, *p* < 0.0001, neurons *t*_(4)_ = 0.4786, *p* = 0.6572; comparisons between glia and neurons: two-tailed independent-samples *t* tests; *Aqp4 t*_(9)_ = 15.99, *p* < 0.0001; *Meg3 t*_(9)_ = 11.46, *p* < 0.0001). *B,* relative expression levels of the mitochondrial genes *Nclx*, *Mcu*, *Micu1*, and *Ppargc1a*, and *Vdac1* (two-tailed independent-samples *t* tests; *Nclx t*_(9)_ = 5.258, *p* = 0.0005, *Mcu t*_(9)_ = 8.220, *p* < 0.0001, *Micu1 t*_(9)_ = 1.738, *p* = 0.1162, *Ppargc1at*_(9)_ = 11.23, *p* < 0.0001, and *Vdac1 t*_(9)_ = 13.08, *p* < 0.0001). *C,* qRT–PCR analysis for sister cultures of mixed, neuronal, and glial cells infected with rAAV-shCTRL or rAAV-shNCLX and harvested on DIV 10. RNA expression levels were normalized to *Gusb*, and then the ratio of shNCLX to shCTRL expression calculated for each gene and culture condition (n = 4–7 independent cultures per condition; one-sample *t* tests *versus* a hypothetical value of one; *Nclx*: mixed *t*_(6)_ = 40.02, *p* < 0.0001, glia *t*_(5)_ = 13.60, *p* < 0.0001, neurons *t*_(4)_ = 9.642, *p* = 0.0006; *Mcu*: mixed *t*_(6)_ = 41.05, *p* < 0.0001, glia *t*_(5)_ = 1.001, *p* = 0.3628, neurons *t*_(4)_ = 11.32, *p* = 0.0003; *Ppargc1a*: mixed *t*_(6)_ = 1.981, *p* = 0.0949, glia *t*_(4)_ = 6.812, *p* = 0.0024, neurons *t*_(4)_ = 0.3786, *p* = 0.7243; *Vdac1*: mixed *t*_(6)_ = 5.136, *p* = 0.0021, glia *t*_(5)_ = 3.432, *p* = 0.0186, and neurons *t*_(4)_ = 3.099, *p* = 0.0363). *D,* primary hippocampal cultures containing both neurons and glia (mixed) or glia-depleted (neurons) or neuron-depleted (glia) sister cultures were infected on DIV 3 with rAAVs driving the expression of either shNCLX or shCTRL and fixed without further treatment on DIV 10 for an assessment of cell viability (n = 4–9 independent cultures; mixed-effects model two-way ANOVA followed by Tukey's multiple comparisons test; main effect of culture type *F*_(2,16)_ = 39.51, *p* < 0.0001; shCTRL: mixed *versus* neurons *q*_(9,8)_ = 8.416, *p* = 0.0076, mixed *versus* glia *q*_(9,4)_ = 6.250, *p* = 0.0317; shNCLX: mixed *versus* neurons *q*_(9,8)_= 8.416, *p* = 0.0076, mixed *versus* glia *q*_(9,4)_ = 6.250, *p* = 0.0317; main effect of shRNA *F*_(1,8)_ = 29.19, *p* = 0.0006; shCTRL *versus* shNCLX: mixed *q*_(9,9)_ = 7.641, *p* = 0.0123; neurons *q*_(8,8)_ = 7.641, *p* = 0.0123), glia *q*_(4,4)_ = 7.641, *p* = 0.0123. ns, ∗*p* < 0.05, ∗∗*p* < 0.01, ∗∗∗*p* < 0.001, and ∗∗∗∗*p* < 0.0001. Bar graphs show the mean + SEM. *Aqp4*, aquaporin 4; DIV, day *in vitro*; *Gusb*, glucoronidase, beta; *Mcu*, mitochondrial uniporter; *Meg3*, maternally expressed gene 3; *Micu1*, mitochondrial calcium uptake 1; NCLX, solute carrier family 8 sodium/calcium/lithium exchanger, member B1; ns, not significant; *Ppargc1a*, peroxisome proliferative activated receptor, gamma, coactivator 1 alpha; qRT–PCR, quantitative reverse transcription polymerase chain reaction; rAAV, recombinant adeno-associated viral vector; shRNA, short hairpin RNA; *Vdac1*, voltage-dependent anion channel 1.

Astrocytes are known to provide metabolic and redox homeostatic, signaling, and structural supports to neurons and are as such important for maintaining their function and survival (63). We therefore questioned whether neuronal loss induced by NCLX knockdown in our primary cultures may be a secondary consequence of mitochondrial dysregulation and associated degeneration of astrocytes rather than a direct consequence of reduced neuronal expression of NCLX. To address this question, we prepared nominally glia-free neuronal and nominally neuron-free glial cultures and compared the effects of NCLX knockdown in these cultures to those observed in mixed hippocampal cultures cultivated using our standard methodology. We confirmed the relative purity of our neuronal cultures infected with rAAV-shCTRL immunocytochemically, wherein we observed an average of 0.78 ± 0.02% GFAP^+^ cells compared with 11.06 ± 1.63% in mixed sister cultures also infected with rAAV-shCTRL (two-tailed paired-samples *t* test; *t*_(4)_ = 6.269, *p* = 0.0033) and *via* qRT–PCR analysis of the astrocyte-specific and neuron-specific genes aquaporin 4 (*Aqp4*) and maternally expressed 3 (*Meg3*) (60, 61, 64), respectively, for both neuronal and glial cultures compared with mixed sister cultures (Fig. 5*A*). As expected, glial cultures were enriched for and neuronal cultures depleted of the astrocytic gene *Aqp4* compared with mixed cultures (Fig. 5*A*). Glia were also depleted of the neuronal gene *Meg3*, whereas neuronal cultures did not express significantly different levels of *Meg3* compared with mixed cultures (Fig. 5*A*). Expression of both *Aqp4* and *Meg3* was significantly different between neuronal and glial cultures (Fig. 5*A*). We also evaluated the expression levels of *Nclx*, *Mcu*, *Micu1*, *Ppargc1a*, and *Vdac1* in both rAAV-shCTRL-infected and rAAV-shNCLX-infected cells (Fig. 5, *B* and *C*). Consistent with previous reports, we observed significant cell type–dependent differences in the relative expression levels of not only *Nclx* and *Vdac1* in our cultures (59, 60, 61, 64) but also in *Mcu* and *Ppargc1a* (Fig. 5*B*). rAAV-shNCLX infection resulted in a significant reduction of *Nclx* expression in all culture types (Fig. 5*C*). Compared with cells infected with rAAV-shCTRL, *Mcu* expression was reduced in mixed and neuronal but not glial cultures; *Ppargc1a* expression increased in glial but not mixed or neuronal cultures; and Vdac1 expression was increased in glial but decreased in mixed and neuronal cultures infected with rAAV-shNCLX (Fig. 5*C*). Taken together, these results indicate that NCLX-directed shRNA expressed under control of the U6 promoter results in reduced *Nclx* expression in both neurons and glia, but that NCLX knockdown disrupts expression of mitochondrial function–related genes in neurons and glia differently.

We next performed an analysis of cell death in nominally glia-free neuronal and neuron-free glial cultures compared with mixed hippocampal cultures cultivated in parallel to determine whether NCLX knockdown–induced neuronal death could be uncoupled from any effects on astrocytes and vice versa. Consistent with the now accepted idea that neuron–glia interactions are essential for neuronal cell homeostasis and survival (63), we detected higher levels of basal cell death in neuronal cultures than in mixed cultures (Fig. 5*D*). Furthermore, NCLX knockdown resulted in a significant increase in the levels of cell death not only in mixed cultures but also in nominally pure neuronal cultures (Fig. 5*D*). For their part, nominally pure glial cultures exhibited very little cell death overall, although a small difference in the proportion of dead cells could be detected in shNCLX-infected compared with shCTRL-infected cultures (Fig. 5*D*). Taken together, these observations suggest that, although NCLX knockdown does influence glial survival, and while neurons are less viable in the relative absence of glia, reduced NCLX expression can trigger neuronal death independent of signaling from nearby astrocytes.

### NCLX knockdown renders synaptic activity neurotoxic

**Figure 6:**
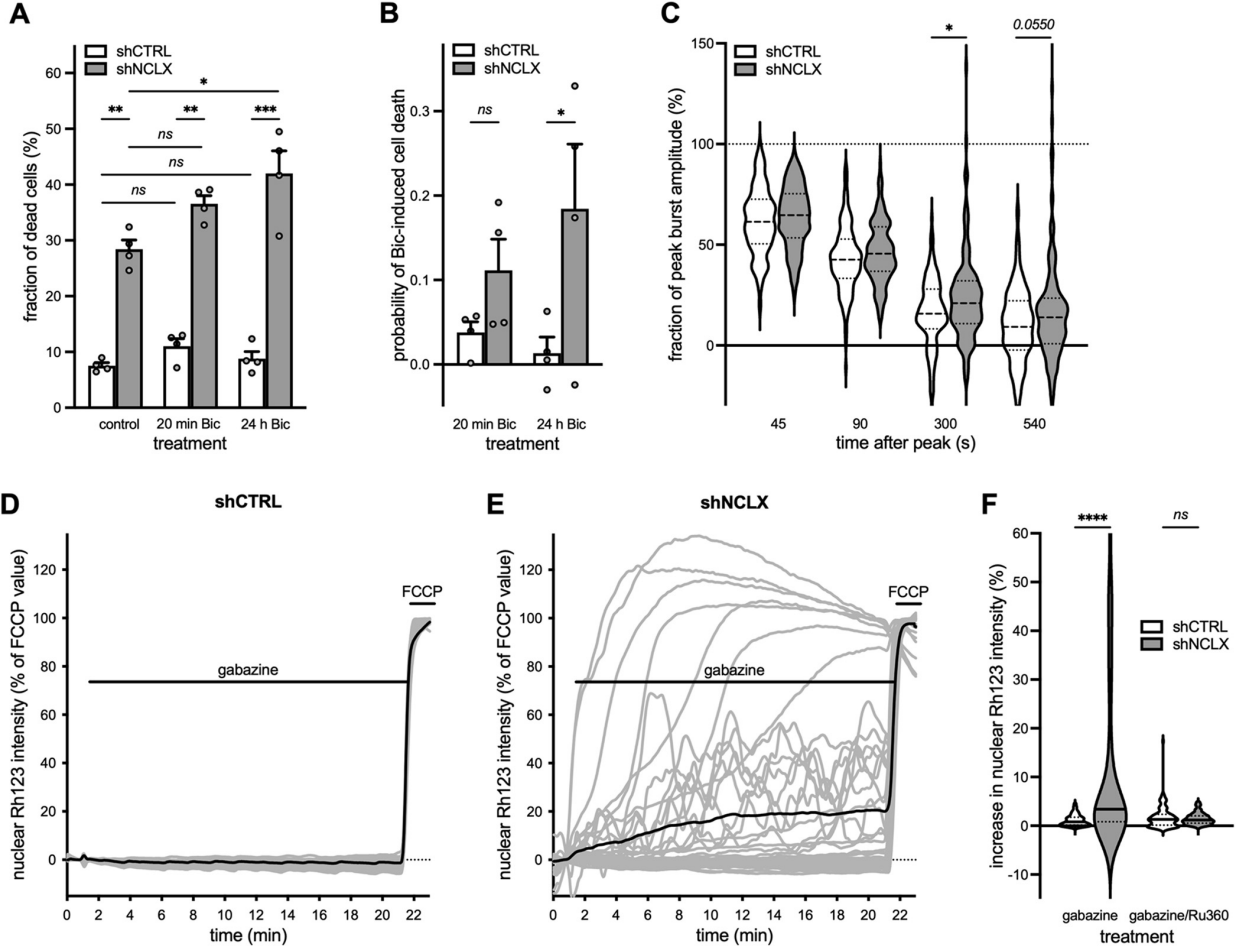
**shRNA-mediated knockdown of NCLX impairs mitochondrial calcium extrusion following action potential bursts, compromises ΔΨ**_**m**_**during neuronal activity, and renders synaptic activity neurotoxic.***A* and *B,* primary hippocampal cultures infected with either rAAV-shCTRL or rAAV-shNCLX were stimulated with the GABA_A_R antagonist, bicuculline (Bic; 50 μM) for 20 min or 24 h to evoke action potential bursting, and then the numbers of live and dead cells assessed after 24 h (n = 4 independent cultures for all conditions). *A,* proportions of dead cells (repeated-measures two-way ANOVA followed by Tukey's multiple comparisons test; main effect of shRNA *F*_(1,3)_ = 283.0, *p* = 0.0005; shCTRL *versus* shNCLX: control *q*_(4,4)_ = 9.219, *p* = 0.0048, 20 min Bic *q*_(4,4)_ = 11.27, *p* = 0.0016, 24 h Bic *q*_(4,4)_ = 14.66, *p* = 0.0004; main effect of treatment *F*_(2,6)_ = 6.042, *p* = 0.0365; control *versus* 20 min Bic: shCTRL *q*_(4,4)_ = 1.543, *p* = 0.8692, shNCLX *q*_(4,4)_ = 3.590, *p* = 0.2454; control *versus* 24 h Bic: shCTRL *q*_(4,4)_ = 0.5523, *p* = 0.9982, and shNCLX *q*_(4,4)_ = 5.990, *p* = 0.0383). *B,* quantification of the probability of cells dying because of Bic treatment, which takes into account elevated levels of basal cell death (see the Experimental procedures section; repeated-measures two-way ANOVA followed by Šídák's multiple comparisons test; shNCLX *versus* shCTRL: 20 min *t*_(4,4)_ = 2.493, *p* = 0.1688, 24 h *t*_(4,4)_ = 5.807, *p* = 0.0202). *C,* the GABA_A_R antagonist, gabazine (5 μM), was used to trigger a single action potential burst in primary hippocampal cultures coinfected with rAAVs driving expression of the mitochondrially targeted FRET-based calcium indicator 4mtD3cpv under control of the CamK2a promoter and either shCTRL or shNCLX. Shown are mitochondrial calcium levels as measured 45, 90, 300, and 540 s after the peak response and normalized to the peak amplitude (shCTRL n = 159 cells from three coverslips and three preparations; shNCLX n = 158 cells from three coverslips and three preparations; Kruskal–Wallis test followed by Dunn's multiple comparisons test; shCTRL *versus* shNCLX: 45 s *Z*_(159,158)_ = 0.6242, *p* = 0.5325, 90 s *Z*_(159,158)_ = 1.186, *p* = 0.2356, 300 s *Z*_(159,158)_ = 2.129, *p* = 0.0333, and 540 s *Z*_(159,158)_ = 1.919, *p* = 0.0550). *D–F,* gabazine (5 μM) was applied in the presence (or not) of the MCU antagonist Ru360 (10 μM) to primary hippocampal cultures infected with rAAVs driving the expression of either shNCLX or shCTRL and loaded with Rh123. When used, Ru360 was present in the culture medium ≥30 min prior to and during the entire course of the experiment. The mitochondrial uncoupler FCCP (5 μM) was used to trigger complete ΔΨ_m_ breakdown. *D* and *E,* representative changes in nuclear Rh123 fluorescence changes to gabazine stimulation in rAAV-shCTRL-infected (*D*) and rAAV-shNCLX-infected cells (*E*) on a single coverslip each (*gray*, individual cells; *black*, their mean). *F,* quantification of changes in ΔΨ_m_ (gabazine: shCTRL n = 194 cells from six coverslips and five preparations, shNCLX n = 171 cells from four coverslips and three preparations; gabazine/Ru360: shCTRL n = 121 cells from three coverslips and three preparations, shNCLX n = 132 cells from four coverslips and three preparations), expressed as the peak amplitude of the nuclear Rh123 intensity during 20 min gabazine stimulation (Kruskal–Wallis test followed by Dunn's multiple comparisons test; shCTRL *versus* shNCLX: gabazine *Z*_(194,171)_ = 8.770, *p* < 0.0001, gabazine + Ru360 *Z*_(121,132)_ = 0.5102, *p* = 0.6099). ns, ∗*p* < 0.05, ∗∗*p* < 0.01, ∗∗∗*p* < 0.001, and ∗∗∗∗*p* < 0.0001. Bar graphs show the mean + SEM. Violin plots show the probability density of the data as well as median and quartile divisions. Bic, bicuculline; CamK2a, calcium/calmodulin dependent protein kinase II alpha; FCCP, carbonyl cyanide-*p*-trifluoromethoxyphenylhydrazone; FRET, Förster resonance energy transfer; GABA_A_R, gamma-aminobutyric acid (GABA) A receptor; MCU, mitochondrial calcium uniporter; NCLX, solute carrier family 8 sodium/calcium/lithium exchanger, member B1; ns, not significant; rAAV, recombinant adeno-associated viral vector; Rh123, rhodamine 123; Ru360, ruthenium 360; ΔΨ_m_, mitochondrial membrane potential.

**Figure 7:**
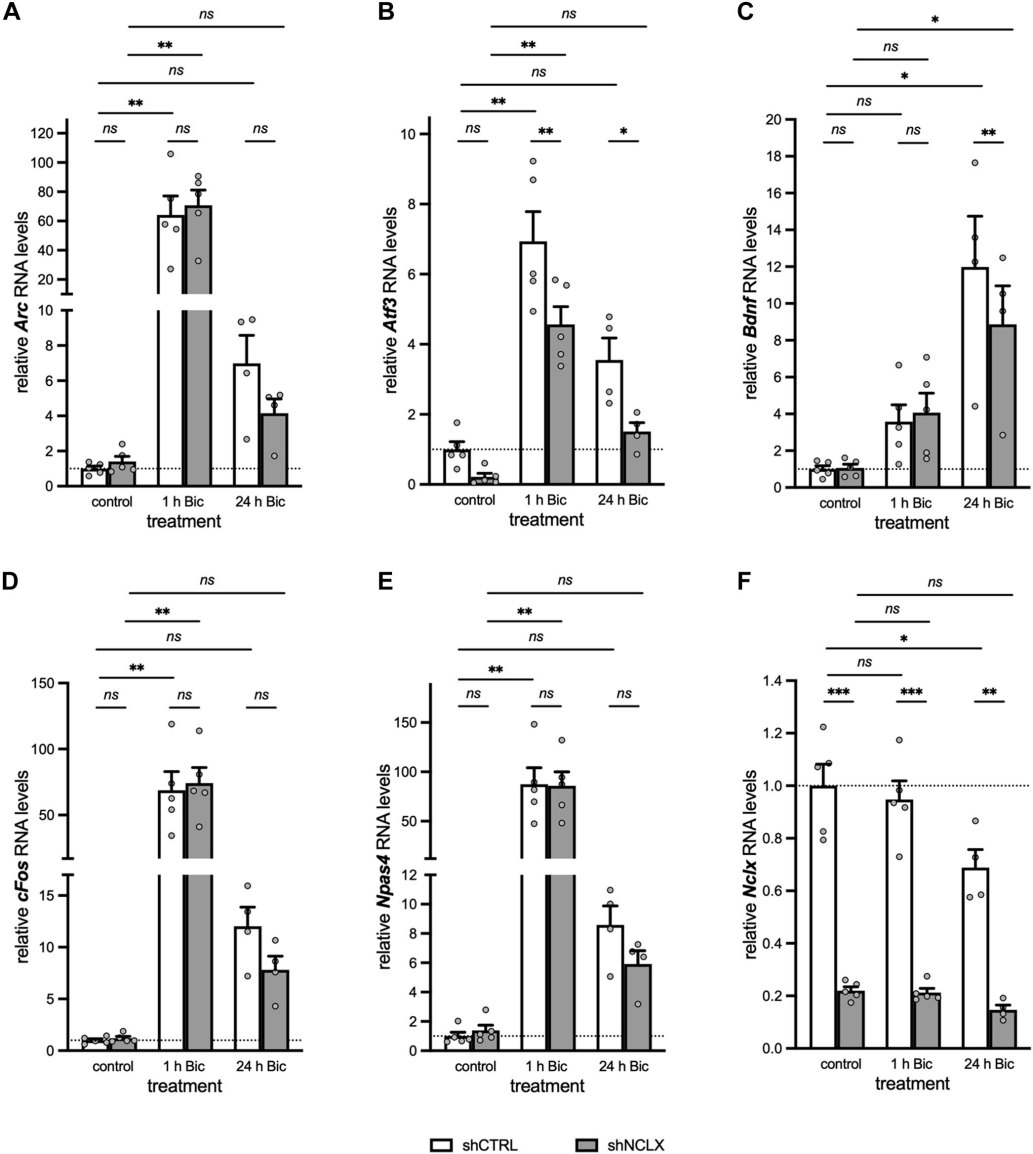
**shRNA-mediated NCLX knockdown does not grossly affect synaptic activity–dependent gene transcription.** QRT–PCR analysis of activity-induced genes *Arc* (*A*), *Atf3* (*B*), *Bdnf* (*C*), *cFos* (*D*), and *Npas4* (*E*), as well as *Nclx* (*F*) for primary hippocampal cultures infected with either rAAV-shCTRL or rAAV-shNCLX and stimulated (or not) with the GABA_A_R antagonist, bicuculline (Bic; 50 μM) for 1 h or 24 h to evoke action potential bursting (n = 4–5 independent cultures; mixed-effects model two-way ANOVAs followed by Tukey's multiple comparisons tests; *A*, *Arc*: main effect of treatment *F*_(2,8)_ = 28.39, *p* = 0.0002, shCTRL control *versus* 1 h Bic *q*_(5,5)_ = 8.871, *p* = 0.0058, shCTRL control *versus* 24 h Bic *q*_(5,4)_ = 0.7924, *p* = 0.9904, shNCLX control *versus* 1 h Bic *q*_(5,5)_ = 9.742, *p* = 0.0036, shNCLX control *versus* 24 h Bic *q*_(5,4)_ = 0.3635, *p* = 0.9998; main effect of shRNA *F*_(1,4)_ = 0.3612, *p* = 0.5802, control shCTRL *versus* shNCLX *q*_(5,5)_ = 0.1479, *p* > 0.9999, 1 h Bic shCTRL *versus* shNCLX *q*_(5,5)_ = 2.427, *p* = 0.5637, 24 h Bic shCTRL *versus* shNCLX *q*_(4,4)_ = 0.9323, *p* = 0.9806. *B, Atf3*: main effect of treatment *F*_(2,8)_ = 32.38, *p* = 0.0001, shCTRL control *versus* 1 h Bic *q*_(5,5)_ = 7.302, *p* = 0.0011, shCTRL control *versus* 24 h Bic *q*_(5,4)_ = 0.9848, *p* = 0.0854, shNCLX control *versus* 1 h Bic *q*_(5,5)_ = 8.907, *p* = 0.0057, shNCLX control *versus* 24 h Bic *q*_(5,4)_ = 2.498, *p* = 0.5424; main effect of shRNA *F*_(1,4)_ = 67.98, *p* = 0.0012, control shCTRL *versus* shNCLX *q*_(5,5)_ = 3.171, *p* = 0.3385, 1 h Bic shCTRL *versus* shNCLX *q*_(5,5)_ = 9.589, *p* = 0.0039, 24 h Bic shCTRL *versus* shNCLX *q*_(4,4)_ = 7.389, *p* = 0.0145. *C, Bdnf*: main effect of treatment *F*_(2,8)_ = 12.26, *p* = 0.0037, shCTRL control *versus* 1 h Bic *q*_(5,5)_ = 1.983, *p* = 0.7269, shCTRL control *versus* 24 h Bic *q*_(5,4)_ = 7.969, *p* = 0.0100, shNCLX control *versus* 1 h Bic *q*_(5,5)_ = 2.318, *p* = 0.6061, shNCLX control *versus* 24 h Bic *q*_(5,4)_ = 5.661, *p* = 0.0488; main effect of shRNA *F*_(1,4)_ = 12.77, *p* = 0.0233, control shCTRL *versus* shNCLX *q*_(5,5)_ = 0.2197, *p* > 0.9999, 1 h Bic shCTRL *versus* shNCLX *q*_(5,5)_ = 1.769, *p* = 0.8005, 24 h Bic shCTRL *versus* shNCLX *q*_(4,4)_ = 9.928, *p* = 0.0032. *D, cFos*: main effect of treatment *F*_(2,8)_ = 23.48, *p* = 0.0004, shCTRL control *versus* 1 h Bic *q*_(5,5)_ = 8.607, *p* = 0.0068, shCTRL control *versus* 24 h Bic *q*_(5,4)_ = 1.320, *p* = 0.9237, shNCLX control *versus* 1 h Bic *q*_(5,5)_ = 9.268, *p* = 0.0069, shNCLX control *versus* 24 h Bic *q*_(5,4)_ = 0.7911, *p* = 0.9285; main effect of shRNA *F*_(1,4)_ = 0.1792, *p* = 0.6938, control shCTRL *versus* shNCLX *q*_(5,5)_ = 0.1527, *p* > 0.9999, 1 h Bic shCTRL *versus* shNCLX *q*_(5,5)_ = 4.251, *p* = 0.1456, 24 h Bic shCTRL *versus* shNCLX *q*_(4,4)_ = 2.973, *p* = 0.3916. *E*, *Npas4*: main effect of treatment *F*_(2,8)_ = 25.40, *p* = 0.0003, shCTRL control *versus* 1 h Bic *q*_(5,5)_ = 9.196, *p* = 0.0048, shCTRL control *versus* 24 h Bic *q*_(5,4)_ = 0.7618, *p* = 0.9920, shNCLX control *versus* 1 h Bic *q*_(5,5)_ = 8.980, *p* = 0.0055, shNCLX control *versus* 24 h Bic *q*_(5,4)_ = 0.4546, *p* = 0.9993; main effect of shRNA *F*_(1,4)_ = 0.8438, *p* = 0.4103, control shCTRL *versus* shNCLX *q*_(5,5)_ = 0.2204, *p* > 0.9999, 1 h Bic shCTRL *versus* shNCLX *q*_(5,5)_ = 0.9754, *p* = 0.9766, 24 h Bic shCTRL *versus* shNCLX *q*_(4,4)_ = 1.419, *p* = 0.9012. *F*, Nclx: main effect of treatment *F*_(2,8)_ = 5.121, *p* = 0.0.0370, main effect of shRNA *F*_(1,4)_ = 251.2, *p* < 0.0001, shCTRL control *versus* 1 h Bic *q*_(5,5)_ = 1.065, *p* = 0.9664, shCTRL control *versus* 24 h Bic *q*_(5,4)_ = 5.678, *p* = 0.0482, shNCLX control *versus* 1 h Bic *q*_(5,5)_ = 0.1500, *p* > 0.9999, shNCLX control *versus* 24 h Bic *q*_(5,4)_ = 1.402, *p* = 0.9054, control shCTRL *versus* shNCLX *q*_(5,5)_ = 17.82, *p* = 0.0001, 1 h Bic shCTRL *versus* shNCLX *q*_(5,5)_ = 16.80, *p* = 0.0002, 24 h Bic shCTRL *versus* shNCLX *q*_(4,4)_ = 11.44, *p* = 0.0015). ns, ∗*p* < 0.05, ∗*p* < 0.01, and ∗∗∗*p* < 0.001. Bar graphs show the mean + SEM. *Arc*, activity regulated cytoskeletal-associated protein; *Atf3*, activating transcription factor 3; *Bdnf*, brain derived neurotrophic factor; Bic, bicuculline; *cFos*, FBJ osteosarcoma oncogene; GABA_A_R, gamma-aminobutyric acid (GABA) A receptor; NCLX, solute carrier family 8 sodium/calcium/lithium exchanger, member B1; *Npas4*, neuronal PAS domain protein 4; ns, not significant; qRT–PCR, quantitative reverse transcription polymerase chain reaction; rAAV, recombinant adeno-associated viral vector.

After a culturing period of 10 or more days, primary hippocampal neurons have established a rich network of synaptic connections, express functional α-amino-3-hydroxy-5-methyl-4-isoxazolepropionic acid/kainate and NMDA receptors, and demonstrate spontaneous synaptic activity and action potential firing (65, 66, 67). It therefore stands to reason that the elevated basal cell death we observed to be associated with NCLX knockdown may result from dysregulated mitochondrial calcium signaling and ΔΨ_m_ breakdown during spontaneous neuronal activity. To address this hypothesis, we first aimed to test whether limiting synaptic activity during the last 2 days of culture may provide some level of neuroprotection to cultures infected with rAAV-shNCLX. In this vein, we cultivated primary hippocampal cells from DIV 8 to DIV 10 in culture medium containing a slightly reduced concentration of potassium ions (K^+^), as we have observed during our calcium imaging studies that the K^+^ concentration employed—which we estimate lowers the resting membrane potential by approximately 4.7 mV compared with control culture medium—reduces spontaneous action potential generation in our culture system (unpublished observations). Although there was no significant statistical interaction between shRNA and culture medium on cell death rates, cultures infected with 2 × 10^9^ viral particles/ml and grown under these conditions did exhibit slightly lower levels of basal death than those grown in control medium (control shCTRL 16.6 ± 2.8%, control shNCLX 56.0 ± 1.9%, reduced K^+^ shCTRL 13.8 ± 2.9%, reduced K^+^ shNCLX 50.9 ± 3.2%; n = 4 for all conditions; repeated-measures two-way ANOVA; main effect of shRNA *F*_(1,3)_ = 234.5, *p* = 0.0006; main effect of culture medium *F*_(1,3)_ = 4.147, *p* = 0.1345; shRNA × culture medium *F*_(1,3)_ = 5.657, *p* = 0.0978). Since the complete blockade of synaptic activity—achieved for instance by cultivation in the voltage-dependent sodium channel antagonist tetrodotoxin—is itself neurotoxic (*e.g.*, (41, 68), and unpublished observations), we next aimed to determine whether enhancement of synaptic activity could exacerbate basal cell death in rAAV-shNCLX-infected cultures. To these ends, we applied the gamma-aminobutyric acid (GABA) A receptor (GABA_A_R) antagonist bicuculline to primary hippocampal cultures to remove tonic inhibition and induce action potential bursting for a period of 20 min or 24 h and fixed the cells for cell death analysis 24 h after antagonist application. As in our previous experiments, rAAV-shNCLX-infected cells exhibited higher levels of cell death than rAAV-shCTRL-infected cells (Fig. 6*A*). Moreover, in rAAV-shNCLX-infected cultures, but not rAAV-shCTRL-infected cultures, 24 h of bicuculline treatment resulted in significantly greater levels of cell death than control (Fig. 6*A*), and the probability of rAAV-shNCLX-infected cells dying was greater in rAAV-shNCLX-infected cells for the 24 h bicuculline treatment (Fig. 6*B*). In parallel to these cell death analyses, we also employed the GABA_A_R antagonist gabazine to examine whether NCLX knockdown could impair the recovery of synaptic activity–associated mitochondrial calcium rises and whether action potential bursting could trigger ΔΨ_m_ breakdown in cells infected with rAAV-shNCLX. Our data demonstrate that NCLX knockdown did inhibit the decay of action potential–induced mitochondrial calcium transients at time points ≥300 s after a single burst (Fig. 6*C*) without affecting the amplitudes of evoked calcium transients (shCTRL increase in *R*_FRET_ = 0.89 ± 0.03, n = 159 cells from three coverslips and three independent preparations; shNCLX increase in *R*_FRET_ = 0.87 ± 0.03, n =158 cells from three coverslips and three independent preparations; two-tailed independent-samples *t* test; *t*_(315)_ = 0.3331, *p* = 0.7393). Moreover, for trains of action potential bursts lasting 20 min, NCLX knockdown was associated with a loss of ΔΨ_m_ that was not observed in rAAV-shCTRL-infected neurons (Fig. 6, *D–F*; Movies S1 and S2). This was made evident *via* quantification of the amplitude of the Rh123 signal during this same time frame (Fig. 6*F*). To confirm that synaptic activity–induced ΔΨ_m_ loss in rAAV-shNCLX-infected neurons resulted from mitochondrial calcium overload, we inhibited mitochondrial calcium influx pharmacologically using the MCU blocker ruthenium 360 (Ru360). Indeed, MCU antagonism with Ru360 prevented the breakdown of ΔΨ_m_ in rAAV-shNCLX-infected neurons during gabazine-triggered action potential bursting (Fig. 6*F*). To determine whether reduced activity-dependent induction of neuroprotective signaling pathways might contribute to cellular demise in cultures infected with rAAV-shNCLX, we in addition assessed the synaptic activity–dependent expression of several immediate early genes: activity regulated cytoskeletal-associated protein (*Arc*), activating transcription factor 3 (*Atf3*), brain derived neurotrophic factor (*Bdnf*), FBJ osteosarcoma oncogene (*cFos*), and neuronal PAS domain protein 4 (*Npas4*) (68). For all genes analyzed, we observed a significant activity-dependent upregulation for both shCTRL-infected and shNCLX-infected cultures (Fig. 7, *A–E*). Furthermore, while our data did not show any difference in basal expression levels, cultures infected with rAAV-shNCLX did exhibit a significantly reduced upregulation of two analyzed genes, *Atf3* and *Bdnf* (Fig. 7, *A–E*). These data indicate that the activity-dependent activation of gene transcription remains largely intact in rAAV-shNCLX-infected cells. Interestingly, in rAAV-shCTRL-infected cells, *Nclx* expression levels decreased after 24 h bicuculline exposure (Fig. 7*F*). In sum, although synaptic activity is widely considered to be neuroprotective (41, 68, 69, 70), our findings suggest that even minor deficits in mitochondrial calcium extrusion during synaptic activity may—in the face of repeated synaptic activation—push neuronal mitochondria toward a pathologically depolarized state that cannot be accommodated for by the simultaneous induction of immediate early and neuroprotective genes. Thus, dysregulated NCLX expression—in addition to making neurons more vulnerable to excitotoxic stimuli—has the potential to render synaptic activity neurotoxic.

### NCLX knockdown *in vivo* is toxic for both neurons and glia

**Figure 8:**
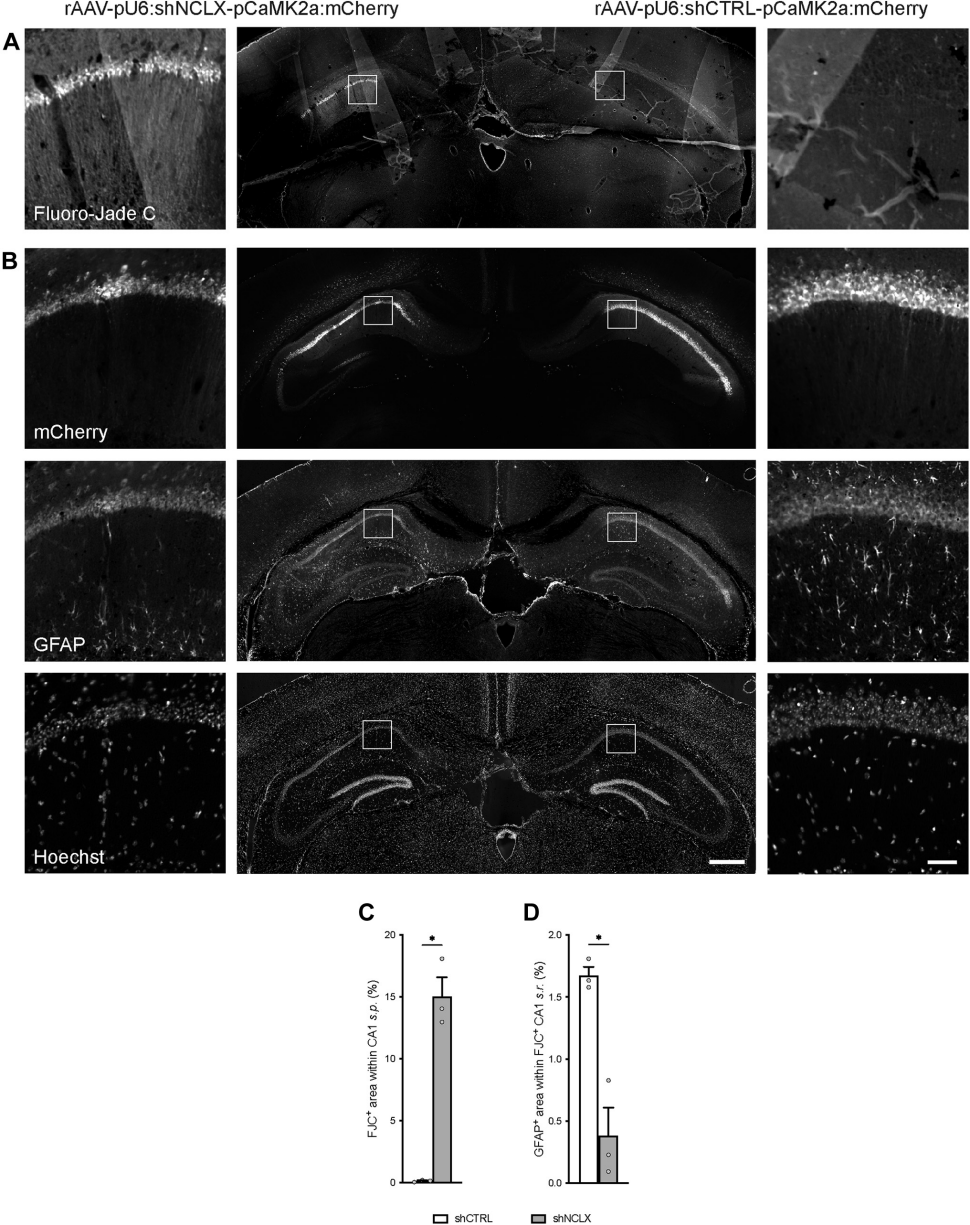
**shRNA-mediated NCLX knockdown leads to loss of neurons and astrocytes *in vivo*.** C57BL/6NCrl mice were stereotactically injected with rAAVs driving the expression of shNCLX in one hemisphere and shCTRL in the other hemisphere (n = 3 mice). *A,* merged image of a representative tissue slice processed using FJC to stain dead and dying neurons. *B,* merged image of the adjacent tissue slice showing mCherry fluorescence, anti-GFAP immunochemistry, and nuclear Hoechst stain. *C,* quantification of the FJC^+^ area within infected regions of dorsal CA1 *s.p.* (two-tailed paired-samples *t* test; *t*_(2)_ = 9.826, *p* = 0.0102). *D,* quantification of the GFAP^+^ area within FJC-labeled regions of CA1 *s.r.* and their counterparts within the contralateral rAAV-shCTRL-infected hemisphere (two-tailed paired-samples *t* test; *t*_(2)_ = 4.535, *p* = 0.0453). The scale bars represent 1 mm (central images) and 50 μm (*insets left and right*). ∗*p* < 0.05. Bar graphs show the mean + SEM. CA1, cornu ammonis 1; FJC, Fluoro-Jade C; GFAP, glial fibrillary acidic protein; NCLX, solute carrier family 8 sodium/calcium/lithium exchanger, member B1; pCaMK2a, calcium/calmodulin-dependent protein kinase II alpha promoter; pU6, U6 small nuclear RNA promoter; rAAV, recombinant adeno-associated viral vector; *s.p.*, stratum pyramidale; *s.r.*, stratum radiatum.

As a final measure, to assess whether NCLX dysregulation could lead to neuron and/or astrocyte loss *in vivo*, we performed a proof-of-principle experiment to assess cellular viability in the CA1 region of the hippocampus of mice that had been injected 4 weeks prior with rAAVs driving the expression of shNCLX in one hemisphere and shCTRL in the contralateral hemisphere (Fig. 8, *A* and *B*). Tissue slices subsequently processed using Fluoro-Jade C (FJC) to stain dead and dying neurons exhibited broad patches of FJC-labeled cells in the hemisphere infected with rAAV-shNCLX but not in the contralateral hemisphere that was infected with rAAV-shCTRL in all three mice (Fig. 8*A*). A quantitative analysis of the area of FJC stain within *stratum pyramidale* (*s.p.*) yielded a significant difference between rAAV-shCTRL-infected and rAAV-shNCLX-infected hemispheres (Fig. 8*C*). Images of mCherry fluorescence from adjacent tissue slices showed that infection rates of rAAV-shNCLX and rAAV-shCTRL were qualitatively similar. Moreover, the density of anti-GFAP immunolabel within *stratum radiatum* (*s.r.*) was significantly reduced (Fig. 8, *B* and *D*), and the numbers of shrunken and dysmorphic nuclei in CA1 *s.p.* markedly increased in the rAAV-shNCLX-infected hemisphere (Fig. 8*B*). Notably, dysmorphic nuclei and loss of GFAP immunoreactivity were predominantly observed in those areas of the hippocampus where, in adjacent slices, FJC-labeled cells could be readily visualized. These data confirm that NCLX knockdown dramatically diminishes neuronal and astrocyte viability under basal conditions *in vivo* and underlines the potential importance of our main findings for neurological diseases associated with dysregulated NCLX expression.

## Discussion

In this study, we investigated the effects of dysregulated expression of the mitochondrial NCLX on neuroglial vulnerability under both excitotoxic conditions and during ongoing synaptic activity. Our results revealed that the shRNA-mediated knockdown of NCLX, as expected, impairs mitochondrial calcium clearance and not only sensitizes neurons to excitotoxic stimuli but also renders synaptic activity toxic. Our results further show that NCLX knockdown diminishes the viability of astroglia. These findings underscore the relevance of intact mitochondrial calcium extrusion mechanisms for determining neuroglial fate not only in the face of excitotoxic challenge but also during otherwise cell survival–promoting synaptic activity.

The data we present here show that impaired NCLX expression rendered neurons more sensitive to excitotoxic stimuli (Figs. 2 and 3). As such, our findings are consistent with several studies in diverse models of neurodegenerative diseases that point to mitochondrial dysfunction as a core trigger for excitotoxic cell death in neurodegenerative disease (3, 4, 5, 6, 7). Our data demonstrate as well that dysregulated NCLX expression is associated with the toxification of synaptic activity (Fig. 6), although neuronal activation of this nature is widely understood to be neuroprotective (41, 68, 69, 70). Transcriptional regulation appears largely intact in cells where NCLX expression was knocked down as synaptic activity was associated with the induction of several immediate early genes (Fig. 7), including ones whose upregulation has been demonstrated to confer neuroprotection (13, 68, 70, 71). Indeed, periodic bursts of synaptic activity are known to activate a nuclear calcium-dependent transcriptional program that enhances neuronal resilience in the face of proapoptotic and excitotoxic stimuli (68, 69, 72). This so-called acquired neuroprotection seems to hinge primarily upon alterations that affect mitochondrial structure and function (8, 72) and includes the transcriptional repression of MCU, which protects cells from not only mitochondrial calcium overload, oxidative damage, and permeability transition (13, 47) but also the upregulation of antioxidant defense genes (73, 74, 75), and a potential metabolic shift in neurons from oxidative phosphorylation toward aerobic glycolysis (72, 76). Our data demonstrate that, while the pathological reduction of NCLX expression does not grossly disrupt activity-dependent gene induction (Fig. 7), evoked—and presumably also basal—synaptic activity is nonetheless rendered toxic (Figs. 4, 6, and 8). These findings lend support to the growing consensus that more work must be done to uncover new therapeutic approaches that limit or prevent mitochondrial calcium overload in acute and chronic neurodegenerative disease.

Our observation that NCLX expression was decreased following extended periods (24 h) of synaptic activity (Fig. 6) may seem, at first thought, to contradict the idea that a consequence of synaptic activity is the reduction of mitochondrial calcium influx and a shift away from calcium-dependent oxidative phosphorylation. Activity-dependent decreases in MCU expression can be seen after as little as 4 h of synaptic activity (13). It is reasonable that a subsequent compensatory decrease in NCLX expression might follow. Clearly, a more thorough time-course analysis of the expression changes of these two integral components of the mitochondrial calcium signaling machinery would provide more insight into the physiological responses of healthy neurons' mitochondria to synaptic activity.

Long-lasting excitotoxic stimuli in rAAV-shCTRL-infected cells in our study evoked mitochondrial calcium rises that failed to recover after removal of the stimulus (Fig. 2). By contrast, even longer-lasting excitotoxic stimuli of the same magnitude failed to elicit a change in the mitochondrial redox potential or ΔΨ_m_ in similarly rAAV-shCTRL-infected cells (Fig. 3). While this disparity between mitochondrial calcium recovery, redox potential changes, and ΔΨ_m_ breakdown in response to an excitotoxic stimulus may at first glance seem surprising given the established links between mitochondrial calcium dysregulation and the mechanisms underlying excitotoxic cell death (1, 2, 3, 4, 5, 6, 7, 8), there is precedent that a sustained cytoplasmic (and presumably also mitochondrial) calcium rise does not necessarily lead to the breakdown of ΔΨ_m_ or to neuronal death (41, 53). Accordingly, observations such as those made here (Figs. 2 and 3) indicate that a prolonged mitochondrial calcium signal may not, *per se*, trigger the loss of physiological mitochondrial function and neuronal cell death. An additional perturbation, such as the strong activation of extrasynaptic NMDAR-linked signaling or—as shown here—the disrupted expression of NCLX, and therewith the loss of intact mitochondrial calcium extrusion mechanisms, is required to render mitochondrial calcium dysregulation toxic. Further investigations clarifying the precise and clearly complex relationships between mitochondrial calcium levels, ΔΨ_m_ breakdown, and redox signaling as well as cell death in the face of both excitotoxic stimuli and synaptic activity are warranted.

NCLX knockdown in our study was accompanied by decreases in the neuronal expression of *Mcu* and *Vdac1* and a trend toward or a decrease in the expression of *Micu1* (Figs. 1 and 5). Endogenous loss of NCLX has been observed in humans and mouse models of AD, where it was similarly accompanied by changes in the levels of the MCU-associated proteins MICU1 and MICUB (25). Given that we could not identify any changes in the expression of *Tfam* or of any analyzed mitochondrial genes (Figs. 1 and 5), it seems unlikely that the downregulated expression of *Mcu*, *Micu1*, or *Vdac1* genes is reflective of neuronal mitophagy. The decreased expression of these genes, the protein products of which control mitochondrial calcium influx, would be expected to result in diminished mitochondrial calcium entry and provide some measure of neuroprotection (5, 13, 20, 36, 47). On the other hand, the decreased expression or activity of NCLX has been associated in some studies with larger amplitude-evoked mitochondrial calcium transients (24, 25, 77). We only observed significant differences in the amplitudes of evoked mitochondrial calcium signals in cells infected with NCLX-targeted shRNA for NMDA stimuli lasting 120 s but not for brief (30 s) or prolonged (300 s) stimuli (Fig. 2). It thus seems possible that the decreased expression of *Mcu*, *Micu1*, and *Vdac1* we observed in cells infected with rAAV-shNCLX may be part of a compensatory response to prolonged mitochondrial calcium transients triggered by NCLX knockdown.

NCLX knockdown in astrocytes was accompanied by the increased expression of *Ppargc1a*, a positive regulator of mitochondrial biogenesis and respiration (31, 32, 33, 78), and *Vdac1*, a gatekeeper for the mitochondria-to-cytoplasm transport of metabolites, including pyruvate (Fig. 5) (34, 35). Together, these changes suggest that astrocytes may compensate for the loss of mitochondrial calcium homeostasis and almost certain metabolic disturbance by an upregulation of mitogenesis. On the other hand, elevated VDAC1 expression and subsequent oligomerization have been proposed to constitute a focal point in apoptotic signaling cascades, also in the context of neurodegenerative disease (34, 35). One might therefore expect an increase in VDAC1 expression to render astrocytes more prone to apoptosis. Alternatively, in light of the fact that astrocytes cultivated in the absence of neurons exhibit an immature phenotype in terms of their gene expression, morphology, and metabolism (79), both the altered mitochondrial gene expression patterns related to and the functional consequences of knocking down NCLX for astrocytic function may be different in astrocytes cultivated under conditions more closely resembling the physiological state. In accordance with this idea, although we found the difference in basal cell death rates of shNCLX-infected compared with shCTRL-infected glial cultures to be statistically significant, the overall rate of shNCLX-associated loss of astrocytes in nominally pure cultures was far lower than that observed for these cells in a mixed culture system (Figs. 4 and 5). It will be exciting to discover in future studies how pathologically reduced NCLX expression and/or function influences neuronal and glial mitogenesis/mitophagy as well as both cell autonomous and intercellular metabolism and metabolic signaling.

Although astroglial functions are impaired in neurodegenerative diseases, and while these impairments are known to involve dysregulated intracellular calcium signaling and mitochondrial function (80, 81, 82), astrocytes have been largely ignored in studies addressing mitochondrial calcium signaling in the context of excitotoxicity and neurodegeneration (63). One exception is a very recent study examining the influence of tau protein on cytosolic and mitochondrial calcium homeostasis (24). In this article, which focused primarily on neurons, the authors demonstrated that treatment of neurons or astrocytes in culture with the K18 repeat domain fragment of tau inhibited the recovery of evoked mitochondrial calcium transients in these cells. On this background, our observation that NCLX knockdown impacts astrocyte viability (Figs. 4 and 8) indicates that these cells may represent a mostly overlooked target of tau pathology in early stages of AD. Indeed, prior to the development of senile plaques and local astrogliosis, AD is associated with astroglial atrophy and asthenia that may both impair metabolic support of neurons and contribute to synapse loss (80, 81, 82). Further detailed studies aimed at specifically examining the consequences of NCLX loss or dysfunction on astrocytes' morphological stability and metabolic capacity will be necessary, however, to understand the implications of these observations for AD and other pathologies.

In this study, we employed viral-mediated delivery of shRNA as a means to experimentally manipulate NCLX expression in neurons and glia (Figs. 1 and 5). Compared with the use of an NCLX knockout, this approach has the advantage that NCLX expression can be reduced at a later stage in development, thus precluding the activation of unpredictable compensatory mechanisms early in development. rAAV infection simultaneously has the disadvantage that transgene—or shRNA—expression levels can exhibit a high degree of cell-to-cell variability (Fig. S1). Because of the lack of appropriate commercially available anti-NCLX antibodies, it was not possible in this study to directly examine NCLX protein expression at the single cell level or to relate such parameters as mitochondrial calcium recovery, ΔΨ_m_ breakdown, or cellular viability to the degree of NCLX knockdown. Our observation that higher rAAV-shNCLX infection rates resulted in lower *Nclx* RNA levels and diminished viability compared with a lower infection rate (Fig. 4) nonetheless supports the idea that degree of NCLX dysregulation may be a key defining factor for determining how a given cell or population of cells will respond to synaptic activity or excitotoxic challenge. Indeed, we believe that the variability of mitochondrial calcium recovery rates (Fig. 2) and ΔΨ_m_ changes (Fig. 3) in our data—particularly in response to shorter-lasting or less intense excitotoxic stimuli—are most likely attributable to cell-intrinsic differences in NCLX knockdown. Future analyses comparing NCLX expression levels and mitochondrial calcium recovery rates or ΔΨ_m_ changes on a cell-by-cell basis will be revealing in this regard.

Endogenously reduced NCLX expression has been implicated in neuronal toxicity in both Friedreich's ataxia and AD (25, 83, 84). Jadiya *et al.* (25) demonstrated, for instance, that downregulated NCLX expression and subsequent changes in mitochondrial calcium handling may contribute to the pathophysiology of AD. In this study, the authors report an uncompensated and profound loss of NCLX expression in the cortex of AD patients and in mouse models of AD and demonstrate that genetic restoration of NCLX expression prevents cognitive decline and AD pathology in the mouse. Cell type–specific effects of dysregulated NCLX expression were not, however, explored (25). The data we present here show that dysregulated NCLX expression is detrimental for the proper functioning and survival not only of neurons but also for the survival of astrocytes (Figs. 4 and 8) and so are in line with previous studies employing siRNA to knock down NCLX expression in this cell type (77). Moreover, our observation that not only the number of astrocytes but also the proportion of viable cells identified as astrocytes was diminished in cultures where NCLX expression was experimentally reduced (Fig. 4) suggests that these cells are particularly sensitive to the perturbed expression of proteins involved in maintaining mitochondrial homeostasis. Given that NCLX is so much more highly expressed by astrocytes than by neurons, and that Jadiya *et al.* (25) observed a near complete loss of NCLX expression in mouse AD models, it seems quite possible that dysregulated mitochondrial calcium signaling in glia contributes to neuronal compromise and functional deficits in AD pathology. Whether and how glial mitochondrial calcium mishandling plays a role in Friedreich's ataxia remains to be explored, but a functional effect seems likely (85).

## Experimental procedures

### Primary hippocampal cultures

Primary dissociated hippocampal cultures were prepared and maintained as described previously (70) from P0-P1 C57BL/6NCrl mice (Charles River Laboratories; Research Resource Identifier [RRID]: IMSR_CRL:27). Briefly, cells were plated at a density of 1.2 to 1.5 × 10^5^ cells/cm^2^ and grown until DIV 8 in neurobasal-A medium (Gibco; catalog no.: 10888022) supplemented with B27 (Gibco; catalog no.: 17504044), 0.5 mM l-glutamine (Sigma–Aldrich; catalog no.: G7513), 1% rat serum (Biowest; catalog no.: S2150), and penicillin–streptomycin (Gibco; catalog no.: 15140122; 50 U/ml). Cytosine arabinoside (AraC) (Sigma–Aldrich; catalog no.: C1768; 2.8 μM) was added on DIV 3 to prevent the proliferation of glial cells. To obtain nominally glia-free cultures, AraC was added 8 to 10 h after plating on DIV 0. A medium change was performed on DIV 8 wherein either 50% of the growth medium was replaced with neurobasal-A medium supplemented with B27 and 0.5 mM glutamine, or the growth medium was completely replaced with medium consisting of a mixture of buffered saline solution (10 mM Hepes, pH 7.4, 114 mM NaCl, 26.1 mM NaHCO_3_, 5.3 mM KCl, 1 mM MgCl_2_, 2 mM CaCl_2_, 30 mM glucose, 1 mM glycine, 0.5 mM C_3_H_3_NaO_3_, and 0.001% phenol red) and phosphate-free Eagle's minimum essential medium (Gibco; catalog no.: 21090-022) (9:1 v/v), supplemented with insulin (7.5 μg/ml), transferrin (7.5 μg/ml), and sodium selenite (7.5 ng/ml) (ITS Liquid Media Supplement; Sigma–Aldrich; catalog no.: I3146) and 50 U/ml penicillin–streptomycin. To obtain nominally neuron-free glial cultures, cells plated at a density of 1.2 × 10^5^ cells/cm^2^ were grown in Dulbecco's modified Eagle's medium (Gibco; catalog no.: 41965-039) containing 10% fetal bovine serum (Gibco; catalog no.: 10270) and 50 U/ml penicillin–streptomycin. On DIV 3, the cells were washed two times with ice-cold PBS, and a full medium change was performed. A 50% medium change was performed on DIV 6 and DIV 8, and AraC was added to the medium on DIV 8 to halt glial proliferation.

### Pharmacology

The following pharmacological agents were used: bicuculline (ENZO Life Sciences; catalog no.: ALX-550-515; 50 μM), CGP37157 (Sigma–Aldrich; catalog no.: C8874; 10 μM), diamide (Santa Cruz Biotechnology; catalog no.: 10465-78-8; 0.5 mM), carbonyl cyanide-*p*-trifluoromethoxyphenylhydrazone (FCCP) (Sigma–Aldrich; catalog no.: C2920; 5 μM), gabazine (SR 99531 hydrobromide; Biotrend; catalog no.: BN0507; 5 μM), NMDA (Biotrend; catalog no.: BN0385; 5, 10, or 20 μM), and Ru360 (Merck Millipore; catalog no.: 557440; 10 μM).

### rAAVs and plasmids

Viral particles were produced and purified as described previously (70, 86). To drive expression of the mitochondrially targeted FRET-based calcium indicator 4mtD3cpv in excitatory neurons, we used a previously described viral vector containing a CaMK2a promoter (13). For expression of shRNA, we used an rAAV vector containing the U6 promoter for shRNA expression and the CaMK2a promoter for expression of mCherry to enable the identification of infected excitatory neurons (13). Two different shRNA sequences against mouse NCLX were generated using iRNAi (Softonic; RRID: SCR_015548), cloned, and tested for silencing efficiency (shNCLX-1, ATGTTGGACTGTGGATCTAAA; shNCLX-2, CGACAAGGACGATCGGAATTG). ShNCLX-1 was selected for most experiments as it provided the most potent knockdown and is also referred to as shNCLX. As a control, we used a sequence with no known targets in the mouse genome (shCTRL, GTGCCAAGACGGGTAGTCA) (27). Unless otherwise stated, rAAV-shCTRL, rAAV-shNCLX-1, and rAAV-shNCLX-2 were used *in vitro* at a concentration of 7 × 10^8^ viral particles/ml. The viral vector driving expression of the mitochondrial matrix–targeted ratiometric glutathione redox potential indicator, glutaredoxin 1-redox-sensitive GFP roGFP2 (mito-Grx1-roGFP2) under control of a cytomegalovirus/chicken beta-actin hybrid promoter, has been described before (47). Primary cultures were infected >6 h after addition of AraC on DIV 3 or DIV 4 (mixed cultures and glia-free cultures) or >6 h after the complete medium change on DIV 3 or DIV 4 (glial cultures). The plasmid used to drive expression of EGFP and murine NCLX (12) under the control of a cytomegalovirus/chicken beta-actin hybrid promoter (pAAV-EGFP.T2A.NCLX) was generated using standard cloning techniques using a previously described mouse NCLX overexpression construct (12). A large portion of the mouse NCLX 3′UTR (nucleotides 1908–2681 from NM_133221) was amplified from mouse genomic DNA using the following primers: 5′-AGTCCCAAGCTTCTGAAGCTGCTTGGCCTAGAGG-3′ and 5′-ATGCCCAAGCTTGGAGGCAAAGGCAGGCAGATTTC-3′ and inserted into a HindIII restriction site downstream from the NCLX coding sequence. All plasmids were verified by sequencing.

### Cell culture

HEK293 cells (Stratagene; catalog no.: 240073) were cultivated in Dulbecco's modified Eagle's medium supplemented with 10% fetal bovine serum, nonessential amino acids (Gibco; catalog no.: 11140-035; 1:100 dilution), sodium pyruvate (Gibco; catalog no.: 11360-039; 1:100 dilution), and 50 U/ml penicillin–streptomycin, and passaged when they reached ∼60 to 70% confluency using typsin/EDTA (Gibco; catalog no.: 25300-054). For immunoblot analyses, cells were plated at a density of 62,600 cells/cm^2^ in a 12-well plate and cotransfected 24 h later with 1 μg pAAV-EGFP.T2A.NCLX and 1 μg pAAV-shCTRL, pAAV-shNCLX, pAAV-shNCLX-2, or none of these, or with 1 μg pAAV-mCherry.NLS or 1 μg pAAV-NCLX.Flag using 2 μl Lipofectamine 2000 Reagent (Invitrogen; catalog no.: 11668-019) following the manufacturer's instructions. Cells were imaged 24 h later using a Vert.A1 upright microscope outfitted with an Axiocam ERc 5s camera and LabScope software (all Zeiss) and then harvested for subsequent immunoblot analysis.

### Fluorescence imaging

All live imaging experiments were performed in a Hepes-buffered saline (HBS) solution containing, in millimolar: 140 NaCl, 2.5 KCl, 1.0 MgCl_2_, 2.0 CaCl_2_, 10 Hepes, 1.0 glycine, 35.6 d-glucose, and 0.5 sodium pyruvate. NMDA-induced and gabazine-induced changes in mitochondrial calcium levels, ΔΨ_m_, and glutathione redox potential were analyzed as described using the FRET-based mitochondrially targeted calcium indicator, 4mtD3cpv, the ratiometric redox potential indicator mito-Grx1-roGFP2, and the small molecule dye, Rh123 (Molecular Probes; catalog no.: R302), respectively (13, 41, 47, 87). Fluorescence images were acquired from cells bathed in room-temperature HBS at 0.667 to 2.0 Hz with a cooled CCD camera (iXon, Andor or ImagEMX2; Hamamatsu) through a 20× water-immersion objective (XLMPlanFluor, 0.95W; Olympus) on an upright microscope (BX51W1; Olympus). Fluorescence excitation (4mtD3cpv: cyan fluorescent protein 430 ± 12 nm, yellow fluorescent protein 500 ± 10 nm; Rh123: 470 ± 20 nm; mito-Grx1-roGFP2: 405 ± 10 and 470 ± 20 nm; AHF Analysentechnik) was provided by a xenon arc lamp in combination with an excitation filter wheel (cellˆR; Olympus). Mito-Grx1-roGFP2 and Rh123 fluorescence was filtered using a 525 ± 25 nm emission filter (AHF Analysentechnik). For 4mtD3cpv imaging, cyan fluorescent protein (470 ± 12 nm) and yellow fluorescent protein (535 ± 15 nm) emission wavelengths were separated and filtered using a DualView beam splitter (AHF Analysentechnik and MAG Biosystems). Data were collected using proprietary software (cellˆR; Olympus) and analyzed using Fiji (RRID: SCR_02283) and IgorPro (WaveMetrics; RRID: 000325). Only morphologically intact cells expressing mCherry were chosen for analysis. Cells with disintegrated dendrites or swollen somata were excluded from analysis. Mitochondrial calcium concentration changes in regions of interest (ROIs) drawn around individual neurons were quantified using the crosstalk- and bleaching-corrected 4mtD3cpv FRET ratio (*R*_FRET_) (88). For ΔΨ_m_ imaging, primary hippocampal cultures were loaded with Rh123 (4.3 μM), a positively charged dye that accumulates inside mitochondria because of their negative intraluminal charge with respect to the cytosol, in HBS for 30 min at room temperature, followed by extensive washing with HBS. When loaded at the concentrations employed in this study, intramitochondrial Rh123 reaches levels that result in self-quenching, such that intramitochondrial Rh123 exhibits less fluorescence that it would were the same amount of dye to be distributed in a markedly larger volume. During ΔΨ_m_ breakdown, Rh123 is released into the cytosol, where—on account of the proportionally much larger volume of this subcellular compartment—self-quenching does not restrict its fluorescence. Since ΔΨ_m_ breakdown–associated Rh123 fluorescence changes originating in mitochondria presumably reflect the combined effects of dequenching and decreased dye load, which would result in increased and decreased fluorescence, respectively, we reasoned that clear signals that reflect a loss of ΔΨ_m_ would be best obtained by measuring cytosolic Rh123 fluorescence. Because of its small molecular size, cytosolic Rh123 can freely diffuse into the nucleus. Accordingly, cytosolic and nuclear Rh123 levels, which are near zero under basal conditions, rise in both compartments upon ΔΨ_m_ breakdown. Thus, intranuclear Rh123 fluorescence increases reliably reflect the ΔΨ_m_ breakdown–associated release of Rh123 from mitochondria. As it is much more straightforward to define mitochondria-free ROIs within the nuclear compartment than within the non-nuclear cytosol, we measured Rh123 fluorescence intensity over time in the nucleus of imaged cells as a readout of depolarizing changes in ΔΨ_m_ (13, 53, 57, 58, 88). Maximum Rh123 signal was obtained at the conclusion of each experiment by exposing the recorded cells to the mitochondrial uncoupler FCCP. Mean nuclear Rh123 fluorescence intensity was quantified on a cell-by-cell basis as a percent of the FCCP-induced level, with baseline (measured in the last 10 s prior to NMDA/gabazine application) set to 0%. Imaging of mito-Grx1-roGFP2 was either performed as aforementioned or at the Nikon Imaging Center (Heidelberg University) using an automated inverted Nikon Ti microscope equipped with a Nikon Plan Fluor 20×/0.75 multi-immersion objective (water immersion was used), a Yokagawa CSU-X1 confocal scanning unit, a Hamamatsu C9100-02 EMCCD camera and a TokaiHit on-stage incubation system. Mito-Grx1-roGFP2 was sequentially excited every 20 s using 405 and 488 nm laser lines, and emission (527 ± 27.5 nm) was collected for both excitation wavelengths. For all confocal imaging experiments, imaging was performed at 37 °C and ambient CO_2_ levels. Maximum roGFP2 signal was obtained by exposing the cells to the thiol-oxidizing reagent diamide. Glutathione redox potential in ROIs drawn around individual neurons was quantified using the 405/488 emission ratio, *R*, and normalized to the diamide-induced maximum of this ratio, *R*_max_: *R*/*R*_max_. The NMDA response was quantified as the amplitude 10 min after NMDA application using the baseline-subtracted *R*/*R*_max_ ratio: *R*/*R*_max_ − *R*/*R*_max_(baseline), where *R*/*R*_max_(baseline) was measured in the last 10 s prior to NMDA application.

### qRT–PCR

RNA was isolated using the RNeasy Plus Mini Kit (Qiagen; catalog no.: 74106) or Quick-RNA MicroPrep kit (Zymo Research; catalog no.: R1050) with additional on-column DNase I digestion according to the manufacturer's instructions. For complementary DNA synthesis, RNA was reverse transcribed with the High-Capacity cDNA Reverse Transcription Kit (Applied Biosystems; catalog no.: 4368813). qRT–PCR was performed on an ABI7300 or StepOnePlus thermal cycler using TaqMan gene expression assays (Applied Biosystems; catalog no.: 4331182) for the following genes: *Aqp4* (Mm00802131_m1), *Arc* (Mm00479619_m1), *Atf3* (Mm00476032_m1), *Bdnf* (exon IV; Mm00432069_m1), *cFos* (Mm00487425_m1), *mCherry* (Mr07319438_mr), *Mcu* (*Ccdc109a*; Mm01168773_m1), *Meg3* (Mm00522599_m1), *Micu1* (Mm00522778_m1), *Nclx* (*Slc8b1*; Mm00519262_m1), *Npas4* (Mm00463644_m1), *Ppargc1a* (Mm01208835_m1), and *Vdac1* (Mm00834272_m1) or using Power SYBR Green reagent (Applied Biosystems; catalog no.: 4367659) with the following primer pairs: *Tfam* (forward: CAGGAGGCAAAGGATGATTC; reverse: CCAAGACTTCATTTCATTGTCG), *mt-Atp6* (forward: ACCTGGTGAACTACGACTGCTAGA; reverse: TGCTTGATTTAGTCGGCCTGGGAT), *mt-Co1* (forward: CTCGCCTAATTTATTCCACTTCA; reverse: GGGGCTAGGGGTAGGGTTAT); *mt-Co2* (forward: CAGTCCCCTCCCTAGGACTT; reverse: TCAGAGCATTGGCCATAGAA); and *mt-Nd1* (forward: GGGATAACAGCGCAATCCTA; reverse: ATCGTTGAACAAACGAACCA) (89). Expression levels of target genes evaluated using TaqMan reagents were normalized to the expression of the housekeeping gene, glucoronidase, beta (*GusB*) (Mm00446953_m1); expression levels of genes evaluated using Power SYBR Green reagent were normalized to the expression of actin beta (*Actb*) (forward: CTAAGGCCAACCGTGAAAAG; reverse: ACCAGAGGCATACAGGGACA). For each independent experiment or set of experiments, basal expression of the untreated rAAV-shCTRL-infected control was set to 100%, and the remaining conditions normalized accordingly.

### Immunocytochemistry and cell death analysis

Cells grown in 4-well or 24-well plates were fixed using Roti-Histofix 4% (Carl Roth; catalog no.: P087) or 4% paraformaldehyde plus 4% sucrose in PBS for 15 min at room temperature and then washed with PBS. When antibody staining was performed, cells were then permeabilized with methanol at −20 °C, blocked using 10% normal goat serum in PBS, and incubated in mouse anti-GFAP antibody diluted 1:500 in 2% bovine serum albumin plus 0.1% Triton X-100 in PBS overnight at 4 °C. The cells were then incubated in 1:500 Dylight488-conjugated donkey antimouse secondary antibody for 1 h at room temperature and mounted in Mowiol 4-88 medium containing 2 μg/ml Hoechst 33258 as a nuclear counterstain. When antibody staining was not performed, cells were mounted after fixation and PBS washes with Mowiol 4-88 containing Hoechst 33258. For robust quantification of glial cells and nuclei, 16 to 20 evenly distributed fields of view were examined per condition and preparation. Images were obtained using a Leica DMIRBE inverted microscope equipped with a 10×/0.3 objective and a SPOT Insight 14 bit CCD camera (Visitron Systems) or a 16 bit Neo sCMOS camera (Andor Technologies) with VisiView imaging software (Visitron Systems) or using a Ti2 Eclipse inverted microscope (Nikon) equipped with a Sola SE II Light Engine (Lumencor), an S Plan Fluor 20×/0.45 objective (Nikon), and a DS-Qi2 14 bit CCD camera (Nikon) with NIS-Elements imaging software (Nikon; RRID: SCR_014329) at the Nikon Imaging Center at Heidelberg University. Counts of GFAP-positive cells were obtained manually for each field of view. Nuclei were identified using custom pipelines in CellProfiler Image Analysis Software (CellProfiler; RRID: SCR_007358). Dead cells, which were characterized by their amorphous or shrunken nuclei as described previously (90), were quantified semiautomatically using CellProfiler Analyst (CellProfiler; RRID: SCR_010649). All conditions for each preparation were analyzed in parallel. The probability of a cell surviving any given treatment can be expressed as the product of the probability that it might survive under basal conditions with the probability that it might survive the treatment: *P*_survival_ = (1 − *P*_basal_) × (1 − *P*_treatment_), where *P*_survival_ is the probability of a cell surviving following a given treatment, *P*_basal_ is probability of a cell dying in the absence of any treatment, and *P*_treatment_ is the probability of a cell dying specifically because of the treatment administered (13). To determine whether NCLX knockdown specifically rendered cultured cells more vulnerable to treatments, we solved the aforementioned equation for *P*_treatment_. For NMDA toxicity experiments, medium was exchanged on DIV 10 with pre-equilibrated medium containing 0, 5, 10, or 20 μM NMDA. Cells were returned to the incubator for 10 min and then washed three times with fresh and equilibrated medium before being returned to the incubator for another 16 to 24 h prior to fixation. The same procedure was followed for experiments involving stimulation with 50 μM bicuculline for 20 min or 24 h.

### Immunoblot analysis

Immunoblotting was carried out using standard procedures. HEK293 cells were harvested directly in sample buffer containing 30% glycerol, 4% SDS, 0.02% bromophenol blue, and 160 mM Tris–HCl, pH 6.8. Antibodies used were rabbit anti-GFP (Molecular Probes; catalog no.: A6455; RRID: AB_221570, 1:20,000 in 5% nonfat milk), mouse anti-α-tubulin (Sigma–Aldrich; catalog no.: T9026; RRID: AB_477593, 1:100,000 in 5% nonfat milk), rabbit anti-Slc8b1 (Thermo Fisher Scientific; catalog no.: PA5-114330; RRID: AB_2890499, 1:500 in 5% bovine serum albumin), horseradish peroxidase–conjugated donkey anti-mouse IgG (H + L) (Dianova; catalog no.: 715-035-150; RRID: AB_2340770, 1:5000 in 5% nonfat milk), and horseradish peroxidase–conjugated goat anti-rabbit IgG (H + L) (Dianova; catalog no.: 111-035-144; RRID: AB_2307391, 1:5000 in 5% nonfat milk). Enhanced chemiluminescence signals were generated using enhanced chemiluminescence reagent (Bio-Rad; catalog no.: 1705061), detected with a Chemidoc Imaging System (Bio-Rad; RRID: SCR_019684), and quantified using Image Studio Lite (RRID: SCR_013715).

### Mice and stereotactic surgery

We used four female C57BL/6NCrl mice (Charles River Laboratories; RRID: IMSR_CRL:27) from 5 to 9 weeks of age. One mouse was excluded from the analysis because the hippocampus was badly damaged during cryoslicing. The mice were group-housed on a 12 h light/dark cycle with food and water *ad libitum*. All procedures were performed according to the German guidelines for the care and use of laboratory animals and with the European Community Council Directive 86/609/EEC and were approved by local authorities.

rAAVs were injected into the dorsal hippocampus using the following coordinates relative to bregma (two injection sites per hemisphere): −2.1 mm anteroposterior, ±1.5 mm mediolateral, −1.4 and −1.6 mm dorsoventral; −2.6 mm anteroposterior, ±2.5 mm mediolateral, and −1.9 and −2.1 mm dorsoventral. A total volume of 2 μl of a 2:1 PBS:20% mannitol solution containing ∼1 × 10^12^ rAAV particles per ml was injected into each hemisphere. The injection speed was 200 nl/min through a 33 Ga needle. The needle was left for 60 or 120 s at each injection site to allow the fluid to diffuse prior to moving or withdrawing the needle, respectively.

### Immunohistochemistry and FJC staining

Four weeks following stereotactic surgeries, mice were anesthetized with an overdose of pentobarbital (300 mg/kg) and then perfused briefly with PBS followed by 4% paraformaldehyde in PBS, pH 7.4, to fix the tissue. Brains were removed and post-fixed in the same solution overnight and then placed into a PBS containing 0.04% thimerosal (Sigma–Aldrich) to prevent contamination and 30% sucrose for cryoprotection. Brain sections (20 μm) were mounted directly onto Superfrost Plus Slides (Thermo Fisher Scientific; catalog no.: J1800AMNZ) and stored at −20 °C until further processing. For immunostaining, slices on slides were permeabilized and blocked in antibody solution consisting of 1:9 normal goat serum and buffer containing 0.2% gelatin, 33 mM Na_2_HPO_4_, 0.6% Triton X-100, and 0.9 M NaCl for 90 min at room temperature. Slices were incubated with mouse anti-GFAP antibody (Cell Signaling Technology; catalog no.: 3670; RRID: AB_561049) diluted 1:300 in antibody solution for 3 days at 4 °C, and then in 1:500 Dylight488-conjugated donkey anti-mouse secondary antibody (Dianova; catalog no.: 715-485-150; RRID: AB_2687442) for 90 min at room temperature. To reduce background fluorescence, slices were finally incubated in 0.1% Sudan Black B (Acros Organics; catalog no.: 4197-25-5) in 70% ethanol for 20 min at room temperature. Slides were mounted in Mowiol 4-88 (Calbiochem; catalog no.: 475904) medium containing 2 μg/ml Hoechst 33258 (Serva; catalog no.: 15090) as a nuclear counterstain. For FJC staining, slices on slides were dehydrated in 80% ethanol containing 1% NaOH for 5 min, washed, and then incubated in 0.06% KMnO_4_ for 15 min to reduce background followed by 0.0002% FJC (Histo-Chem, Inc; catalog no.: 2FJC) in 1% acetic acid for 30 min. Slices were then washed with water and allowed to dry overnight. They were finally dehydrated in xylene and mounted using Roti-Histokitt II (Carl Roth; catalog no.: T160). Images were obtained with NIS-Elements imaging software (RRID: SCR_014329) at the Nikon Imaging Center at the Heidelberg University using a Ni Eclipse upright microscope equipped with a 10×/0.45 Plan Apo objective and a DS-Qi2 14 bit CCD camera (all Nikon) or using a Leica DMIRBE inverted microscope equipped with a 10×/0.3 objective a 16 bit Neo sCMOS camera (Andor Technologies) with VisiView imaging software (Visitron Systems), and stitched together using the automated Photomerge function of Adobe Photoshop. FJC and GFAP signals were quantified with Fiji software (RRID: SCR_002285). Threshold level was determined from the rAAV-shCTRL-infected hemisphere and set as the mean +3 SD from manually drawn ROIs. For FJC analysis, ROIs encompassed the entire mCherry^+^ dorsal CA1 *stratum pyramidale* in each hemisphere. For GFAP analysis, ROIs were drawn within FJC^+^ areas of *stratum radiatum* in the rAAV-shNCLX-infected hemisphere and mirrored (with minor adjustments) onto to the rAAV-shCTRL-infected hemisphere. Particles were defined as suprathreshold areas exceeding 50 pixels in size. The total area of FJC^+^ or GFAP^+^ particles was obtained from three to four sections for each animal.

### Experimental design and statistical analysis

Statistical analyses were carried out and graphs generated using GraphPad Prism (GraphPad Software, Inc; RRID: SCR_002798). Bar graphs show the mean + SEM. Violin plots show the probability density of the data as well as median and quartile divisions. Outliers were identified using the ROUT method with *Q* = 1%, and data were assessed for normality using the Shapiro–Wilk test. The following statistical tests were used as indicated in the text and figure legends: two-tailed one-sample *t* test *versus* a hypothetical value of 1, Kruskal–Wallis test followed by Dunn's multiple comparisons test, two-tailed paired-samples *t* test, two-tailed independent-samples *t* test, two-tailed independent-samples Mann–Whitney test, mixed-effects model one-way ANOVA followed by Dunnett's multiple comparisons test, mixed-effects model one-way ANOVA followed by Šidák's multiple comparisons test, ordinary one-way ANOVA followed by Šídák's multiple comparisons test, ordinary two-way ANOVA followed by Tukey's multiple comparisons test, ordinary two-way ANOVA followed by Šídák's multiple comparisons test, repeated-measures two-way ANOVA followed by Tukey's multiple comparisons test, repeated-measures two-way ANOVA followed by Šídák's multiple comparisons test, mixed-effects model two-way ANOVA followed by Tukey's multiple comparisons test, and mixed-effects model two-way ANOVA followed by Šídák's multiple comparisons test. Precise *p* values are provided in the text or figure legends, as appropriate, and significance levels are indicated on figures as follows: not significant (ns), ∗*p* < 0.05, ∗∗*p* < 0.01, ∗∗∗*p* < 0.001, and ∗∗∗∗*p* < 0.0001.

## Data availability

All data generated during the experimental procedures in this article are contained within the article and/or are available on request.

## Supporting information

This article contains supporting information.

## Conflict of interest

The authors declare that they have no conflicts of interest with the contents of this article.
