# Supplementary material for: Disrupted expression of mitochondrial NCLX sensitizes neuroglial networks to excitotoxic stimuli and renders synaptic activity toxic
Source: J Biol Chem. 2021 Dec 20;298(2):101508. doi: 10.1016/j.jbc.2021.101508 (PMC8808183; doi:10.1016/j.jbc.2021.101508)
Supplement: Supporting information [file mmc3.pdf]

Supporting Information

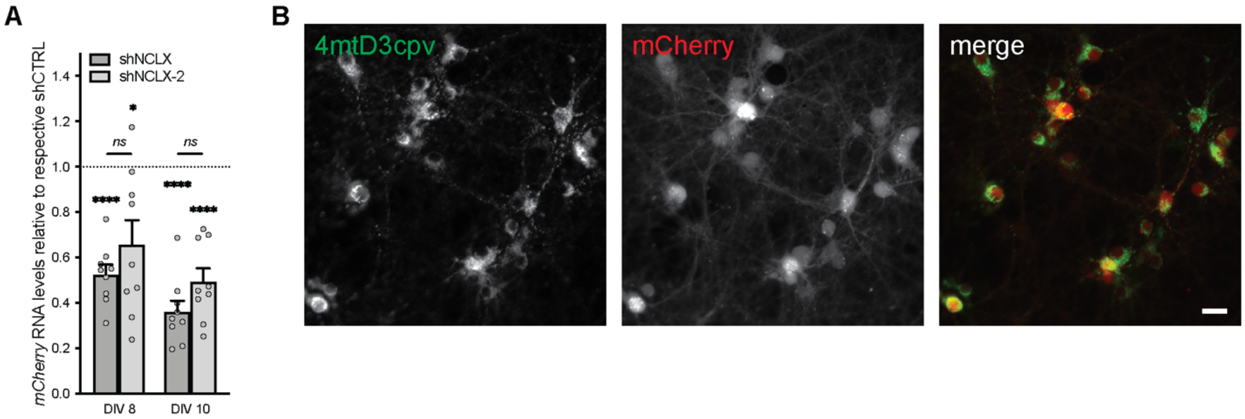

Figure S1. rAAV-mediated *mCherry* expression in primary hippocampal cultures. *A*, Primary hippocampal cultures were infected on DIV 3 with with rAAVs driving the expression of shCTRL, shNCLX, or shNCLX-2 under control of the U6 promoter along with mCherry as an infection marker under control of the CaMK2a promoter as shown in Fig. 1*A*. qRT–PCR analysis of the *mCherry* message (normalized to *Gusb* and expressed as a fraction of the levels in rAAV-shCTRL-infected cultures) on five and seven days after infection, on DIV 8 and DIV 10 (n = 9 independent cultures; two-tailed one-sample t-tests vs. a hypothetical value of 1; DIV 8: shNCLX $t_{(8)} = 11.03$, $p < 0.0001$, shNCLX-2: $t_{(8)} = 3.208$, $p = 0.0125$; DIV 10: shNCLX $t_{(8)} = 13.04$, $p < 0.0001$, shNCLX-2: $t_{(8)} = 8.776$, $p < 0.0001$; ordinary 1-way ANOVA followed by Šidák's multiple comparisons test; shNCLX vs. shNCLX-2: DIV 8 $t_{(9,9)} = 1.375$, $p = 0.3256$, DIV 10 $t_{(9,9)} = 1.379$, $p = 0.3235$). *B*, Representative wide-field image of primary hippocampal neurons coinfected with rAAV-4mtD3cpv and rAAV-shNCLX. The expression levels of both 4mtD3cpv and the rAAV-shNCLX infection marker mCherry exhibit substantial cell-to-cell variability. Scale bar: 20 µm. *ns*, **p* < 0.05, *****p* < 0.0001. Bar graphs show the mean + SEM. DIV, day *in vitro*; CaMK2a, calcium/calmodulin-dependent protein kinase II alpha; *Gusb*, glucoronidase, beta; NCLX, solute carrier family 8 sodium/calcium/lithium exchanger, member B1; ns, not significant; qRT–PCR, quantitative reverse transcription polymerase chain reaction; rAAV, recombinant adeno-associated viral vector.

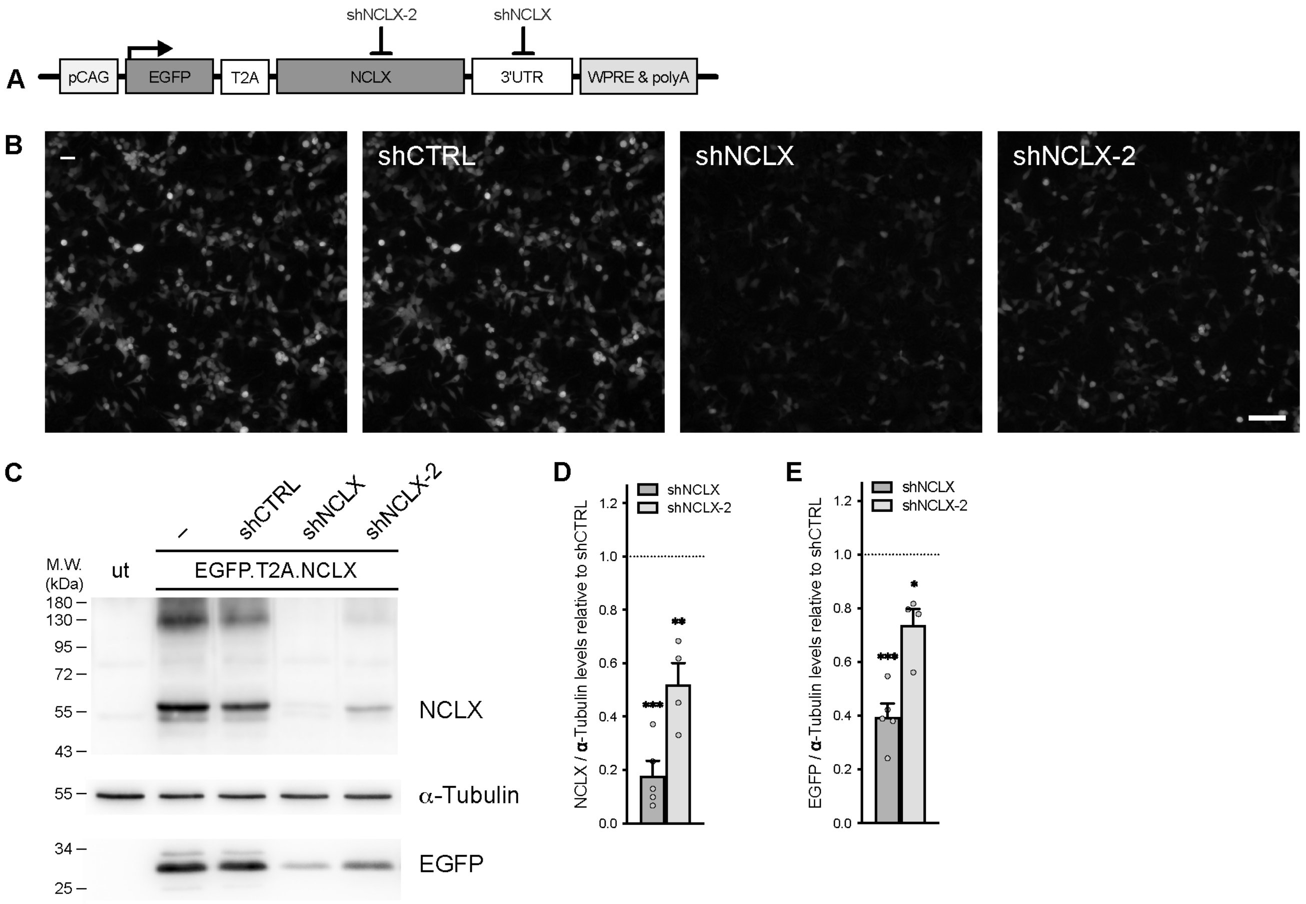

Figure S2. shNCLX and shNCLX-2 reduce target expression at the protein level. *A*, Design of a plasmid construct driving the co-expression of EGFP and mouse NCLX separated by the T2A self-cleaving peptide (pAAV-EGFP.T2A.NCLX). Also shown are the target sites for shNCLX-2 within the NCLX protein coding sequence and shNCLX within the mouse NCLX 3′ untranslated region (3′UTR). HEK293 cells were co-transfected with pAAV-EGFP.T2A.NCLX and pAAV-shCTRL (shCTRL), pAAV-shNCLX (shNCLX), pAAV-shNCLX-2 (shNCLX-2), or none of these (–). *B*, Representative images showing EGFP fluorescence in HEK293 cells 24 h after transfection. *C*, Representative immunoblot analysis of untransfected (ut) HEK293 cells and of HEK293 cells cotransfected as above and harvested 24 h later. *D*, Relative expression levels of NCLX (quantification of the doublet centered on 55 kDa; normalized to α-Tubulin) (n = 4–5 independent cultures; two-tailed one-sample t-tests vs. a hypothetical value of 1; shNCLX $t_{(4)} = 14.79$, $p = 0.0001$, shNCLX-2 $t_{(3)} = 5.994$, $p = 0.0093$. *E*, Relative expression levels of EGFP (normalized to α-Tubulin) (n = 4–5 independent cultures; two-tailed one-sample t-tests vs. a hypothetical value of 1; shNCLX $t_{(4)} = 12.35$, $p = 0.0002$, shNCLX-2 $t_{(3)} = 4.40$, $p = 0.0217$. Scale bar: 100 µm. *$p < 0.05$, **$p < 0.01$, ***$p < 0.001$. Bar graphs show the mean + SEM. 3′UTR, 3′ untranslated region; EGFP, enhanced GFP; HEK293, human embryonic kidney 293 cells; M.W., molecular weight; NCLX, solute carrier family 8 sodium/calcium/lithium exchanger, member B1; pCAG, CAG promoter; qRT–PCR, quantitative reverse transcription polymerase chain reaction; rAAV, recombinant adeno-associated viral vector; ut, untransfected; WPRE, woodchuck hepatitis virus posttranscriptional regulatory element.

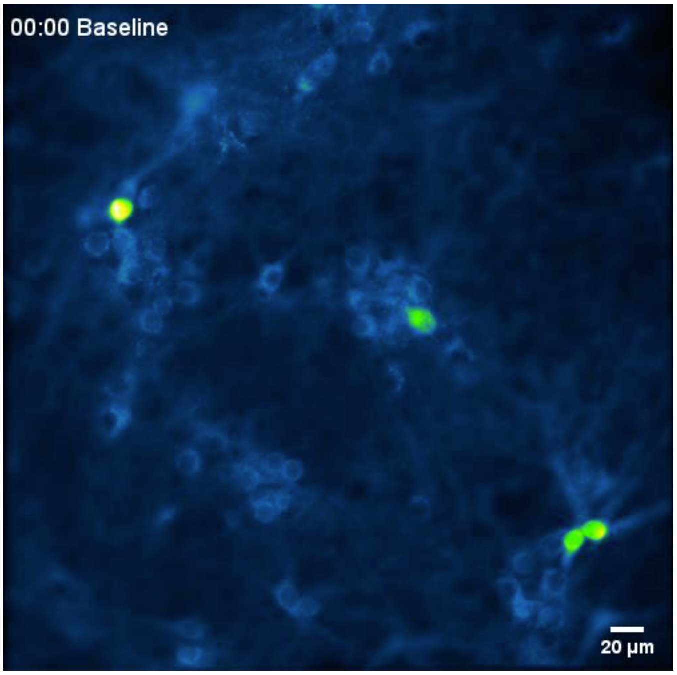

Movie S1. Representative Rh123 fluorescence during gabazine treatment in rAAV-shCTRL-infected neurons. Gabazine (5 µM) and FCCP (5 µM) were applied at the times indicated. Scale bar: 20 µm. FCCP, carbonyl cyanide-*p*-trifluoromethoxyphenylhydrazone; rAAV, recombinant adeno-associated viral vector; Rh123, rhodamine 123.

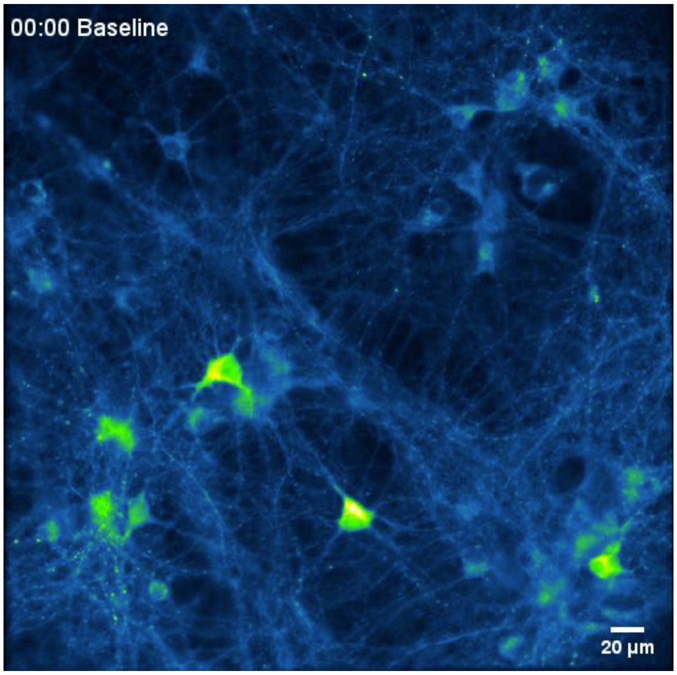

Movie S2. Representative Rh123 fluorescence during gabazine treatment in rAAV-shNCLX-infected neurons. Gabazine (5 µM) and FCCP (5 µM) were applied at the times indicated. Scale bar: 20 µm. FCCP, carbonyl cyanide-*p*-trifluoromethoxyphenylhydrazone; rAAV, recombinant adeno-associated viral vector; Rh123, rhodamine 123.
